# Association between exposure to per- and polyfluoroalkyl substances and kidney function: a population study

**DOI:** 10.3389/fmed.2025.1569031

**Published:** 2025-03-26

**Authors:** Xue Zhang, Yongping Cao, Xiaona Yang, Fei Ma, Hengyang Zhang, Wenwen Xiao

**Affiliations:** ^1^School of Pharmaceutical Sciences, Zhejiang Chinese Medical University, Hangzhou, China; ^2^Eastern Theater Command Centers for Disease Control and Prevention, Nanjing, China; ^3^Linping District Center for Disease Control and Prevention, Linping District Health Supervision Institute, Hangzhou, Zhejiang, China

**Keywords:** polyfluoroalkyl chemicals, kidney function, eGFR, mixed exposure, National Health and Nutrition Examination Survey

## Abstract

**Background:**

The relationship between per- and polyfluoroalkyl substances (PFAS) and kidney function markers remains uncertain.

**Methods:**

We used PFAS detection data from 5,947 adults in NHANES 2005–2012. We employed multivariable linear regression models to examine associations between PFAS and estimated glomerular filtration rate (eGFR), urine creatinine (UCR), urine albumin (UAL), and urine albumin/creatinine ratio (UACR). To capture non-linear trends, restricted cubic splines were applied. The WQS (weighted quantile sum) and Q-gcomp (quantile g computation) models were used for the mixture analysis. Subgroup analyses were conducted to explore potential interactions.

**Results:**

Perfluorooctanoic acid (PFOA), perfluorooctane sulfonic acid (PFOS), perfluorohexane sulfonic acid (PFHxS), 2-(N-methyl-perfluorooctane sulfonamido) acetic acid (N-MEFOSAA), and perfluorononanoic acid (PFNA) were negatively related to eGFR (*β* = −2.04, 95% CI = −2.85, −1.23; *β* = −0.97, 95% CI = −1.78, −0.16; *β* = −1.50, 95% CI = −2.24, −0.76; *β* = −0.49, 95% CI = −1.25, 0.27; *β* = −0.68, 95% CI = −1.46, 0.10). PFOA and PFOS were positive associated with UCR (*β* = 10.61, 95% CI = −1.89, 23.11; *β* = 12.98, 95% CI = 0.56, 25.41). PFOA, PFOS, PFHxS, PFNA, and PFUA were negatively related to UAL (*β* = −0.53, 95% CI = −0.73, −0.32; *β* = −0.39, 95% CI = −0.59, −0.18; *β* = −0.59, 95% CI = −0.78, −0.40; *β* = −0.42, 95% CI = −0.65, −0.19; *β* = −0.04, 95% CI = −0.22, 0.14). PFDA, PFOA, PFOS, PFHxS, and PFNA are significantly inversely associated with UACR (*β* = −0.01, 95% CI = −0.16, 0.14; *β* = −0.52, 95% CI = −0.69, −0.35; *β* = −0.50, 95% CI = −0.67, −0.33; *β* = −0.49, 95% CI = −0.64, −0.33; *β* = −0.27, 95% CI = −0.44, −0.10). Nonlinear relationships were found between PFAS and all kidney function indicators. Mixed PFAS exposure showed a negative association with eGFR, UAL and UACR, while showed a positive relationship with UCR. Interactions between PFASs and most subgroups were observed.

**Conclusion:**

Our study revealed significant associations between PFAS exposure and various kidney function indicators. These findings provide an epidemiological perspective on how PFAS may lead to kidney dysfunction.

## 1 Introduction

The estimated Glomerular Filtration Rate (eGFR) refers to the amount of fluid filtered through the glomeruli each minute. It reflects the kidney’s filtering capacity and can be accurately estimated using the CKD-EPI formula (1). Urinary creatinine (UCR) is the concentration of creatinine in urine. Creatinine is a product of muscle metabolism and is usually excreted by the kidneys (2). Urinary albumin (UAL) refers to the albumin present in urine and is also an important indicator for evaluating kidney health. eGFR, UCR, and UAL are important biomarkers for assessing kidney function. These markers are essential for diagnosing, monitoring, and predicting kidney diseases. Variations in these markers are strongly associated with both chronic kidney disease (CKD) and acute kidney injury. The World Health Organization (WHO) estimates that around 850 million people globally suffer from some form of kidney disease (3). Millions of deaths each year are due to kidney disease. This issue impacts global health and imposes significant economic burdens on families and society (4). Therefore, monitoring kidney function indicators and preventing kidney diseases is crucial.

Many risk factors influence changes in eGFR and urinary creatinine. These factrors include age, sex, race, diabetes, hypertension, unhealthy lifestyles (such as smoking and drinking), obesity, and environmental factors (5–7). Polyfluoroalkyl chemicals (PFASs) are a type of environmental chemical that has received attention in recent years. They consist of synthetic compounds that contain fluorine and carbon. Their molecular structure typically includes one or more fluorinated carbon chains (8, 9). PFASs are highly resistant to heat and chemicals and are waterproof. Thus, they are widely used in various industrial and consumer products, such as waterproof coatings, food packaging materials, cleaners, and firefighting foams (10). PFASs are often referred to as “forever chemicals” due to their resistance to degradation, allowing them to persist in the environment for extended periods and potentially posing long-term risks to both ecosystems and human health (11, 12). Humans can be exposed to PFASs through drinking water, food, air, and consumer products. Many populations in various countries have been found to have PFASs in their blood (13–15). Currently, PFASs represent a major public health issue worldwide and have garnered widespread attention. Studies show that PFASs are closely related to various health problems, including endocrine disruption, reproductive issues in both men and women, liver damage, immune system effects, cardiovascular diseases, and certain types of cancer (16–18). Kidneys are the primary pathway for metabolizing PFASs, it may take years for PFASs to be cleared once they enter the kidneys. Long-term accumulation of PFASs in the human body can result in numerous serious effects. Research indicates that exposure to PFASs can lead to kidney enlargement, cause pathological changes in tissues, and alter the permeability of renal microvascular endothelial cells (19). Animal models have shown that exposure to PFASs can cause changes in kidney weight and serum composition (20). Toxicological studies report that PFASs can induce kidney cancer through multiple signaling pathways, evading apoptosis, altering proliferation, and preventing differentiation (21). Epidemiological studies have found a significant association between PFAS levels in the population and the risk of kidney stones (22). Reports indicate that increased serum PFAS levels are significantly associated with uric acid levels, which can increase the risk of hyperuricemia (5). However, there is currently limited direct research on the relationship between PFASs and kidney function indicators. Their association remains unclear.

In previous studies, renal function assessment relied heavily on limited biomarkers, and studies often failed to systematically evaluate the relationships between multiple renal function indicators. We have provided a more comprehensive evaluation framework through comprehensive analysis of renal function indicators, including eGFR, urine creatinine (UCR), urine albumin (UAL), and albumin creatinine ratio (UACR) etc. This not only helps to understand the potential mechanisms of PFAS, but also provides new biomarkers for subsequent clinical and environmental health research. Our research provides valuable public health insights by highlighting the potential impact of PFAS exposure on kidney function in the general population. By establishing a link between PFAS exposure and kidney markers, this study offers a basis for future studies aimed at reducing the health risks associated with environmental pollutants, ultimately contributing to improved population health outcomes.

## 2 Materials and methods

### 2.1 Study populations

The NHANES, conducted by the Centers for Disease Control and Prevention, plays a key role in evaluating the health and nutritional status of the U.S. population. Since the 1960s, the survey has been administered annually, using a nationally representative sample. The NHANES study has received approval from the NCHS Institutional Review Board, and all participants provided written informed consent.

Our research obtained data from years that included measurements of polyfluoroalkyl chemicals. From 2005 to 2012, there were 40,790 NHANES participants. A total of 34,843 participants were excluded from the analysis. This includes 32,433 participants with missing information on polyfluoroalkyl chemicals, 1,750 individuals under the age of 20, and 101 individuals with missing serum and urine creatinine, along with other missing covariate information. The detailed inclusion–exclusion process is presented in Supplementary Figure S1.

### 2.2 Measurement of serum polyfluoroalkyl chemicals

The National Center for Environmental Health measured perfluorinated compounds (PFASs) in serum samples from four NHANES cycles: 2005–2006, 2007–2008, 2009–2010, and 2011–2012. PFASs were detected using solid-phase extraction coupled with high-performance liquid chromatography–tandem mass spectrometry (HPLC-MS/MS). In brief, after dilution with formic acid, a 50 μL serum aliquot is injected into a commercial column-switching system, allowing the analytes to be concentrated on a solid-phase extraction column. Separation of the analytes from other serum components is achieved by high-performance liquid chromatography. Detection and quantification are performed using negative ion turbo spray ionization, a method that enables rapid detection of PFAS in human serum with a detection limit in the parts-per-billion (ppb or ng/mL) range. The NHANES quality assurance and quality control (QA/QC) program complies with the requirements of the Clinical Laboratory Improvement Amendments (CLIA) of 1988 (23). NHANES measured 12 types of PFASs from 2005 to 2012. For further analysis, we selected 7 PFASs with detection rates greater than 50%, including perfluorooctanoic acid (PFOA), perfluorooctane sulfonic acid (PFOS), perfluorohexane sulfonic acid (PFHxS), 2-(N-methyl-perfluorooctane sulfonamido) acetic acid (N-MEFOSAA), perfluorodecanoic acid (PFDA), perfluorononanoic acid (PFNA), and perfluoroundecanoic acid (PFUA). For results below the limit of detection (LOD), values were recorded as LOD divided by the square root of 2.

### 2.3 Outcomes and covariates

Renal function indicators were used as outcomes in this study: eGFR and urine UCR, analyzed as continuous variables. eGFR was calculated using the CKD-EPI creatinine equation based on participants’ serum creatinine, age, and gender. The specific formulas are:


Male:eGFR=141×minScr/0.9,1−0.411×maxScr/0.9,1−1.209×0.993^Age×1.159ifblack.

Female:eGFR=141×minScr/0.7,1−0.329×maxScr/0.7,1−1.209×0.993^Age×1.018×1.159ifblack.

UCR, UAL, and UACR can help reflect the level of renal function. They were measured in urine using the Roche/Hitachi modular P chemical analyzer. UACR is the urine albumin/creatinine ratio.

Covariates were included based on previous studies related to renal function. These covariates included age, gender, race, education level, family income ratio (calculated by dividing the household [or individual] income by the corresponding poverty line), alcohol consumption (ever have 4/5 or more drinks every day), smoking status (have you smoked at least 100 cigarettes in your entire life?), hypertension, diabetes, and body mass index (BMI). Hypertension and diabetes status were determined from self-reported physician diagnoses in structured questionnaires.

### 2.4 Statistical analysis

For continuous variables, we reported the mean and standard deviation, while for categorical variables, we presented counts and percentages. The distribution information and results for PFAS were described using minimum, maximum, and quartiles. Since the distribution of PFAS levels is not normal, the natural logarithm transformation was applied during the correlation analysis.

To assess the association between kidney function and PFAS, we applied a natural logarithm transformation to PFAS. We used multiple linear regression to estimate the linear relationship, presenting the results as *β* values and 95% confidence intervals. The value of *β* represents the change in the outcome variables (eGFR, UCR, UAL, and UACR) for a one-unit increase in the concentration of perfluoroalkyl chemicals. Model 1 adjusted for age, gender, race, education level, and family income ratio. Model 2 further adjusted for smoking information, alcohol consumption, hypertension, diabetes, and BMI.

We fitted the nonlinear relationship between kidney function indicators and PFAS using restricted cubic splines, with 3 knots. The x-axis represented the natural logarithm of PFAS, and the y-axis represented the effect estimates for kidney function indicators.

Mixed exposure is often considered more reflective of real-life situations. Therefore, we used the WQS model and Q-gcomp model to assess the effects of PFAS mixed exposure on kidney function indicators. The bidirectional WQS model calculated the contribution level of each PFAS to the overall effect. The Q-gcomp model estimated the contribution of individual PFASs and the linear relationship between mixed PFASs and kidney function indicators.

Next, we performed subgroup analyses to compare associations between different subgroups. We grouped based on age (<55, ≥50), gender (male, ≥female), race (Non-Hispanic white, others), education level (less than high school, others), smoking status (yes, no), alcohol consumption (yes, no), hypertension (yes, no), diabetes (yes, no), and BMI (<30, ≥30).

All statistical analyses were conducted using R software (version 4.2.1). A *P* value <0.05 was considered statistically significant. The “gWQS” and “qgcomp” packages were used for mixed exposure analysis.

## 3 Results

### 3.1 Population characteristics

The characteristics of the study population were presented in Table 1. After the inclusion and exclusion process, a total of 5,947 participants were included in the analysis of the association between PFAS and kidney function indicators. The average age of the participants was 48.91 ± 17.96 years, and 51.3% were female. The highest proportion of participants were Non-Hispanic White, accounting for 47.9%. The largest group among the participants had a college or AA degree, with 1,716 individuals (28.9%). The proportion of drinkers was high, while the proportion of smokers was low. The hypertension history was reported by 33.7% of participants, and 11% had a history of diabetes. The proportion of overweight and obese individuals was greater than that of those with normal weight. The average eGFR and UCR were 105.28 (16.37) and 124.49 (79.17), respectively.

**Table 1:** Characteristics of the populations.

Characteristic	*N* = 5,947
Age, mean (SD)	48.91 (17.96)
Gender, *n* (%)	
Male	2,898 (48.7)
Female	3,049 (51.3)
Race, *n* (%)	
Mexican American	945 (15.9)
Non-Hispanic Black	1,199 (20.2)
Non-Hispanic White	2,846 (47.9)
Other Hispanic	509 (8.6)
Other Race	448 (7.5)
Education, *n* (%)	
9–11th grade	908 (15.3)
College graduate or above	1,346 (22.7)
High school graduate/GED or equivalent	1,308 (22.0)
Less than 9th grade	655 (11.1)
Some college or AA degree	1716 (28.9)
Unknown	4 (0.1)
Family income ratio, mean (SD)	2.54 (1.63)
Alcohol, *n* (%)	
Yes	3,950 (66.4)
No	1,544 (26.0)
Unknown	453 (7.6)
Smoke, *n* (%)	
Yes	2,719 (45.7)
No	3,225 (54.2)
Unknown	3 (0.1)
Hypertension, *n* (%)	
Yes	2005 (33.7)
No	3,933 (66.1)
Unknown	9 (0.2)
Diabetes, *n* (%)	
Yes	652 (11.0)
No	5,177 (87.1)
Unknown	118 (2.0)
BMI, mean (SD)	28.90 (6.77)
<25	1809 (30.4)
25–30	1973 (33.2)
≥30	2,165 (36.4)
eGFR, mean (SD)	105.28 (16.37)
UCR, mean (SD)	124.49 (79.17)
UAL, mean (SD)	48.80 (475.83)
UACR, mean (SD)	489.28 (4508.75)

SD, standard deviation; AA, associate’s degree; BMI, body mass index; eGFR, estimated glomerular filtration rate; UCR, urine creatinine; UAL, urine albumin; UACR, urine albumin/creatinine ratio.

### 3.2 Distribution of serum PFAS

The distribution of PFASs in serum was presented in Supplementary Table S1, including minimum, maximum, and quartiles values. The detection rates for PFOA (99.68%), PFOS (99.73%), PFHxS (98.68%), and PFNA (99.43%) were all over 95%. The detection rate for PFUA (54.70%) was the lowest among the group. The Spearman correlation coefficients between PFASs varied widely, ranging from 0.06 to 0.74 (Supplementary Figure S2).

### 3.3 Linear associations between single PFAS and kidney function indicators

The association between PFAS quartiles and eGFR is shown in Table 2. The models adjusted for demographic characteristics and other covariates. In the fully adjusted Model 2, we found that the highest quartile concentrations of PFOA, PFOS, PFHxS, N-MEFOSAA, and PFNA showed significant negative changes compared to the lowest quartile (*β* = −2.04, 95% CI = −2.85, −1.23, *P* for trend <0.001; *β* = −0.97, 95% CI = −1.78, −0.16, *P* for trend <0.001; *β* = −1.50, 95% CI = −2.24, −0.76, *P* for trend <0.001; *β* = −0.49, 95% CI = −1.25, 0.27, *P* for trend = 0.008; *β* = −0.68, 95% CI = −1.46, 0.10, *P* for trend = 0.021). No association was observed between other PFASs and eGFR estimates. Table 3 displayed the association between PFAS quartiles and UCR. The results revealed that the highest quartile concentrations of PFOA and PFOS exhibited significantly positive changes compared to the lowest quartile (*β* = 10.61, 95% CI = −1.89, 23.11, *P* for trend <0.001; *β* = 12.98, 95% CI = 0.56, 25.41, *P* for trend <0.001). In contrast, the highest quartile concentrations of PFUA showed significantly negative changes compared to the lowest quartile, (*β* = −8.86, 95% CI = −18.74, 1.02, *P* for trend = 0.009). Table 4 presents the relationship between PFAS and UAL. In the fully adjusted model, we found significantly negative associations between the highest quartiles of PFOA, PFOS, PFHxS, PFNA, and PFUA compared to the lowest quartile with urine albumin (*β* = −0.53, 95% CI = −0.73, −0.32, *P* for trend <0.001; *β* = −0.39, 95% CI = −0.59, −0.18, *P* for trend = 0.002; *β* = −0.59, 95% CI = −0.78, −0.40, *P* for trend <0.001; *β* = −0.42, 95% CI = −0.65, −0.19, *P* for trend = 0.001; *β* = −0.04, 95% CI = −0.22, 0.14, *P* for trend = 0.003). In Table 5, compared to the lowest quartile, the highest quartile levels of PFDA, PFOA, PFOS, PFHxS, and PFNA are significantly inversely associated with UACR (*β* = −0.01, 95% CI = −0.16, 0.14, *P* for trend = 0.040; *β* = −0.52, 95% CI = −0.69, −0.35, *P* for trend <0.001; *β* = −0.50, 95% CI = −0.67, −0.33, *P* for trend <0.001; *β* = −0.49, 95% CI = −0.64, −0.33, *P* for trend <0.001; *β* = −0.27, 95% CI = −0.44, −0.10, *P* for trend <0.001).

**Table 2:** Multiple linear regression between individual PFASs and eGFR.

Variables	Q1	Q2	Q3	Q4	*P* for trend
PFDA					
Model 1	Ref	0.19 (−0.13, 0.51)	0.06 (−0.35, 0.46)	−0.37 (−1.07, 0.32)	0.677
Model 2	Ref	0.16 (−0.16, 0.47)	0.00 (−0.40, 0.40)	−0.39 (−1.09, 0.30)	0.518
PFOA					
Model 1	Ref	−1.35 (−1.89, −0.81)	−1.88 (−2.44, −1.32)	−2.10 (−2.90, −1.29)	<0.001
Model 2	Ref	−1.32 (−1.86, −0.78)	−1.84 (−2.40, −1.27)	−2.04 (−2.85, −1.23)	<0.001
PFOS					
Model 1	Ref	−0.69 (−1.25, −0.14)	−1.38 (−1.96, −0.80)	−0.97 (−1.78, −0.15)	<0.001
Model 2	Ref	−0.66 (−1.21, −0.11)	−1.37 (−1.95, −0.79)	−0.97 (−1.78, −0.16)	<0.001
PFHxS					
Model 1	Ref	−0.72 (−1.23, −0.20)	−1.22 (−1.74, −0.69)	−1.53 (−2.27, −0.79)	<0.001
Model 2	Ref	−0.68 (−1.19, −0.16)	−1.18 (−1.71, −0.66)	−1.50 (−2.24, −0.76)	<0.001
N-MEFOSAA					
Model 1	Ref	−0.38 (−0.69, −0.08)	−0.51 (−0.92, −0.11)	−0.40 (−1.16, 0.37)	0.012
Model 2	Ref	−0.38 (−0.69, −0.07)	−0.52 (−0.92, −0.11)	−0.49 (−1.25, 0.27)	0.008
PFNA					
Model 1	Ref	−0.03 (−0.63, 0.56)	−0.25 (−0.85, 0.35)	−0.74 (−1.53, 0.04)	0.017
Model 2	Ref	−0.03 (−0.61, 0.56)	−0.25 (−0.85, 0.35)	−0.68 (−1.46, 0.10)	0.021
PFUA					
Model 1	Ref	0.23 (−0.09, 0.54)	0.06 (−0.36, 0.48)	0.25 (−0.40, 0.90)	0.330
Model 2	Ref	0.18 (−0.14, 0.49)	0.01 (−0.41, 0.44)	0.26 (−0.39, 0.92)	0.430

Model 1: adjusted for age, sex, race, education level, and family income ratio. Model 2: adjusted plus alcohol drinking, smoke status, diabetes, hypertension, body mass index category based on Model 1. Ref, reference. Ref, reference. Q, quartile.

**Table 3:** Multiple linear regression between individual PFASs and UCR.

Variables	Q1	Q2	Q3	Q4	*P* for trend
PFDA					
Model 1	Ref	1.02 (−3.82, 5.86)	−1.93 (−8.04, 4.17)	−3.33 (−13.86, 7.20)	0.426
Model 2	Ref	2.00 (−2.81, 6.82)	−0.10 (−6.18, 5.99)	−1.73 (−12.18, 8.73)	0.822
PFOA					
Model 1	Ref	5.20 (−3.22, 13.61)	13.69 (4.94, 22.44)	9.93 (−2.65, 22.50)	<0.001
Model 2	Ref	5.64 (−2.72, 14.00)	13.82 (5.12, 22.53)	10.61 (−1.89, 23.11)	<0.001
PFOS					
Model 1	Ref	7.79 (−0.72, 16.30)	15.01 (6.08, 23.94)	11.86 (−0.67, 24.38)	<0.001
Model 2	Ref	7.83 (−0.62, 16.28)	15.58 (6.72, 24.44)	12.98 (0.56, 25.41)	<0.001
PFHxS					
Model 1	Ref	−6.71 (−14.66, 1.24)	−1.80 (−9.99, 6.39)	−4.22 (−15.76, 7.31)	0.474
Model 2	Ref	−6.70 (−14.59, 1.19)	−1.14 (−9.27, 7.00)	−3.05 (−14.50, 8.39)	0.299
N-MEFOSAA					
Model 1	Ref	3.56 (−1.11, 8.23)	1.32 (−4.83, 7.47)	−2.15 (−13.74, 9.44)	0.778
Model 2	Ref	4.43 (−0.21, 9.07)	2.91 (−3.20, 9.01)	−0.18 (−11.69, 11.33)	0.399
PFNA					
Model 1	Ref	4.96 (−4.08, 14.00)	7.01 (−2.16, 16.17)	3.94 (−8.01, 15.90)	0.301
Model 2	Ref	4.72 (−4.25, 13.70)	6.89 (−2.21, 15.99)	4.17 (−7.69, 16.04)	0.268
PFUA					
Model 1	Ref	−5.32 (−10.11, −0.53)	−8.54 (−14.93, −2.14)	−10.61 (−20.55, −0.68)	<0.001
Model 2	Ref	−3.86 (−8.64, 0.93)	−6.46 (−12.84, −0.08)	−8.86 (−18.74, 1.02)	0.009

Model 1: adjusted for age, sex, race, education level, and family income ratio. Model 2: adjusted plus alcohol drinking, smoke status, diabetes, hypertension, body mass index category based on Model 1. Ref, reference. Q, quartile.

**Table 4:** Multiple linear regression between individual PFASs and UAL.

Variables	Q1	Q2	Q3	Q4	*P* for trend
PFDA					
Model 1	Ref	−0.16 (−0.24,-0.08)	−0.17 (−0.26,-0.07)	−0.12 (−0.30,0.06)	0.002
Model 2	Ref	−0.12 (−0.20,-0.04)	−0.10 (−0.20,0.00)	−0.04 (−0.23,0.14)	0.110
PFOA					
Model 1	Ref	−0.39 (−0.53,-0.26)	−0.43 (−0.57,-0.28)	−0.56 (−0.76,-0.36)	<0.001
Model 2	Ref	−0.42 (−0.56,-0.27)	−0.42 (−0.57,-0.27)	−0.53 (−0.73,-0.32)	<0.001
PFOS					
Model 1	Ref	−0.24 (−0.38,-0.11)	−0.30 (−0.44,-0.16)	−0.42 (−0.62,-0.22)	<0.001
Model 2	Ref	−0.27 (−0.41,-0.13)	−0.29 (−0.44,-0.15)	−0.39 (−0.59,-0.18)	0.002
PFHxS					
Model 1	Ref	−0.37 (−0.49,-0.24)	−0.52 (−0.65,-0.39)	−0.58 (−0.76,-0.40)	<0.001
Model 2	Ref	−0.45 (−0.58,-0.32)	−0.56 (−0.69,-0.43)	−0.59 (−0.78,-0.40)	<0.001
N-MEFOSAA					
Model 1	Ref	0.01 (−0.07,0.08)	−0.01 (−0.11,0.09)	0.12 (−0.06,0.30)	0.601
Model 2	Ref	0.03 (−0.05,0.10)	0.02 (−0.09,0.12)	0.18 (0.00,0.37)	0.186
PFNA					
Model 1	Ref	−0.37 (−0.56,-0.19)	−0.47 (−0.66,-0.28)	−0.41 (−0.63,-0.18)	<0.001
Model 2	Ref	−0.38 (−0.57,-0.18)	−0.45 (−0.65,-0.26)	−0.42 (−0.65,-0.19)	0.001
PFUA					
Model 1	Ref	−0.15 (−0.23,-0.07)	−0.21 (−0.31,-0.11)	−0.10 (−0.27,0.07)	<0.001
Model 2	Ref	−0.12 (−0.20,-0.04)	−0.17 (−0.27,-0.06)	−0.04 (−0.22,0.14)	0.003

Model 1: adjusted for age, sex, race, education level, and family income ratio. Model 2: adjusted plus alcohol drinking, smoke status, diabetes, hypertension, body mass index category based on Model 1. Ref, reference. Q, quartile.

**Table 5:** Multiple linear regression between individual PFASs and UACR.

Variables	Q1	Q2	Q3	Q4	*P* for trend
PFDA					
Model 1	Ref	−0.17 (−0.24,-0.10)	−0.16 (−0.25,-0.07)	−0.01 (−0.17,0.14)	0.011
Model 2	Ref	−0.15 (−0.22,-0.08)	−0.13 (−0.21,-0.04)	−0.01 (−0.16,0.14)	0.040
PFOA					
Model 1	Ref	−0.44 (−0.56,-0.32)	−0.54 (−0.66,-0.42)	−0.56 (−0.73,-0.39)	<0.001
Model 2	Ref	−0.42 (−0.53,-0.30)	−0.50 (−0.62,-0.39)	−0.52 (−0.69,-0.35)	<0.001
PFOS					
Model 1	Ref	−0.29 (−0.41,-0.17)	−0.40 (−0.53,-0.28)	−0.51 (−0.69,-0.33)	<0.001
Model 2	Ref	−0.30 (−0.42,-0.18)	−0.40 (−0.52,-0.28)	−0.50 (−0.67,-0.33)	<0.001
PFHxS					
Model 1	Ref	−0.30 (−0.41,-0.19)	−0.46 (−0.58,-0.35)	−0.49 (−0.65,-0.32)	<0.001
Model 2	Ref	−0.33 (−0.43,-0.22)	−0.48 (−0.59,-0.37)	−0.49 (−0.64,-0.33)	<0.001
N-MEFOSAA					
Model 1	Ref	−0.03 (−0.09,0.04)	−0.03 (−0.12,0.06)	0.12 (−0.05,0.29)	0.865
Model 2	Ref	−0.02 (−0.09,0.04)	−0.03 (−0.11,0.06)	0.16 (0.00,0.33)	0.646
PFNA					
Model 1	Ref	−0.27 (−0.40,-0.14)	−0.38 (−0.51,−0.25)	-0.25 (−0.42,-0.08)	<0.001
Model 2	Ref	−0.27 (−0.39,-0.14)	−0.37 (−0.50,-0.24)	−0.27 (−0.44,-0.10)	<0.001
PFUA					
Model 1	Ref	−0.12 (−0.19,-0.05)	−0.12 (−0.22,-0.03)	0.02 (−0.12,0.17)	0.028
Model 2	Ref	−0.09 (−0.16,-0.02)	−0.11 (−0.20,-0.02)	0.03 (−0.11,0.17)	0.069

Model 1: adjusted for age, sex, race, education level, and family income ratio. Model 2: adjusted plus alcohol drinking, smoke status, diabetes, hypertension, body mass index category based on Model 1. Ref, reference. Q, quartile.

We further conducted multiple linear regression analysis with PFASs as continuous variables, as shown in Supplementary Table S2. There was a significant negative correlation between PFOA, PFOS, PFHxS, N-MEFOSAA, and eGFR (*β* = −0.24, 95% CI = −0.44, −0.05, *P* < 0.01; *β* = −0.27, 95% CI = −0.43, −0.11, *P* < 0.001; *β* = −0.22, 95% CI = −0.36, −0.08, *P* = 0.003; *β* = −0.20, 95% CI = −0.34, −0.06, *P* = 0.005). Increased PFOA, PFOS, N-MEFOSAA, and PFNA in serum were significantly positively associated with higher UCR (*β* = 0.06, 95% CI = 0.04, 0.09, *P* < 0.001; *β* = 0.07, 95% CI = 0.05, 0.09, *P* < 0.001; *β* = 0.03, 95% CI = 0.01, 0.05, *P* < 0.001; *β* = 0.04, 95% CI = 0.02, 0.07, *P* < 0.001). Additionally, PFOA, PFOS, PFHxS, and PFNA are significantly negatively associated with UAL (*β* = −0.25, 95% CI = −0.30, −0.20, *P* < 0.001; *β* = −0.12, 95% CI = −0.16, −0.07, *P* < 0.001; *β* = −0.19, 95% CI = −0.23, −0.16, *P* < 0.001; *β* = −0.10, 95% CI = −0.14, −0.05, *P* < 0.001). Regarding UACR, there is a significant negative correlation with PFDA, PFOA, PFOS, PFHxS, and PFNA (*β* = −0.04, 95% CI = −0.08, −0.01, *P* = 0.023; *β* = −0.29, 95% CI = −0.33, −0.24, *P* < 0.001; *β* = −0.17, 95% CI = −0.20, −0.13, *P* < 0.001; *β* = −0.19, 95% CI = −0.22, −0.16, *P* < 0.001; *β* = −0.13, 95% CI = −0.18, −0.09, *P* < 0.001). These results are generally consistent with the findings mentioned above.

### 3.4 Non-linear relationship between single PFAS and kidney function indicators

**Figure 1:**
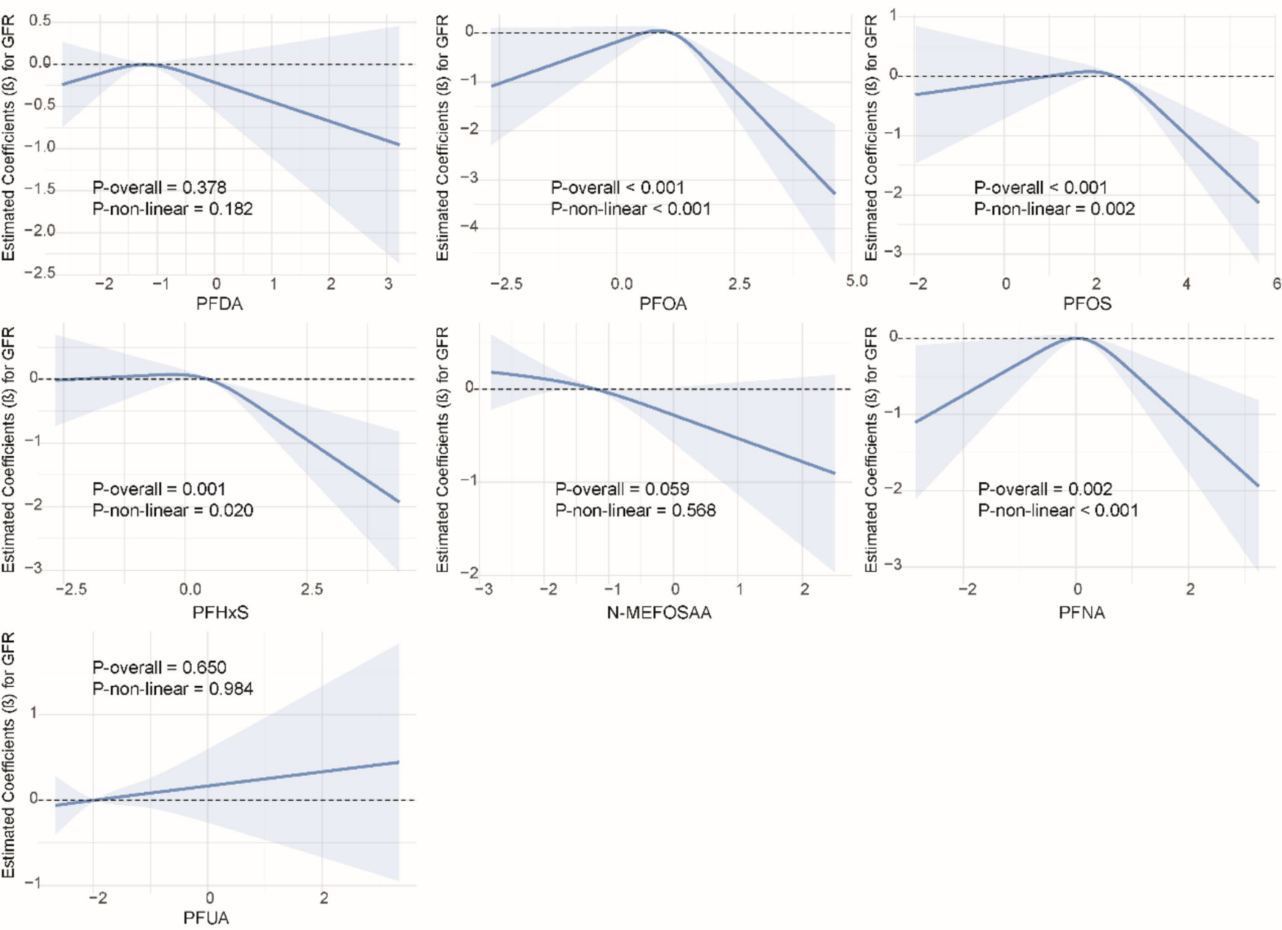
Non-linear dose–response relationships between individual PFASs and eGFR.

**Figure 2:**
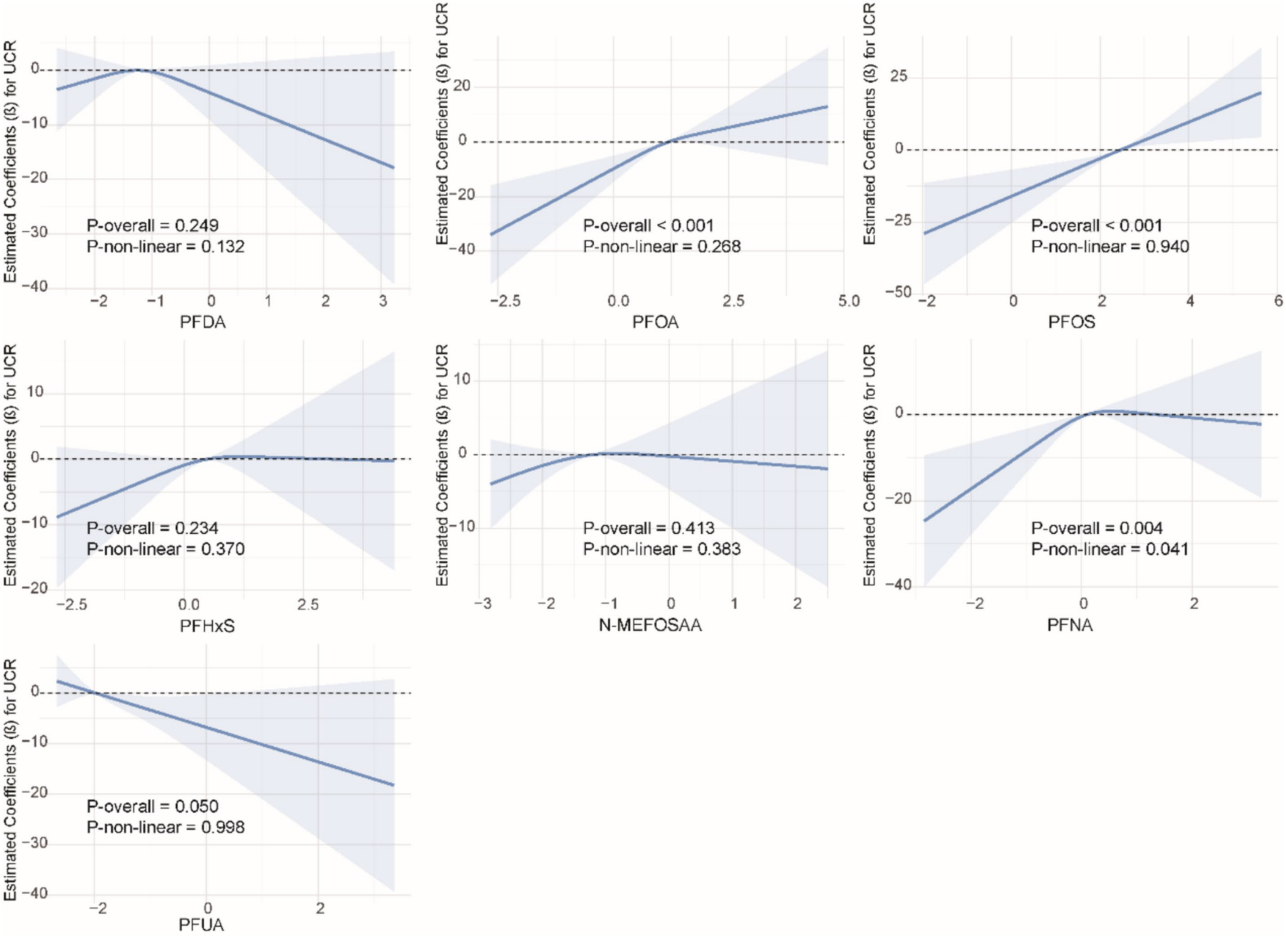
Non-linear dose–response relationships between individual PFASs and UCR.

**Figure 3:**
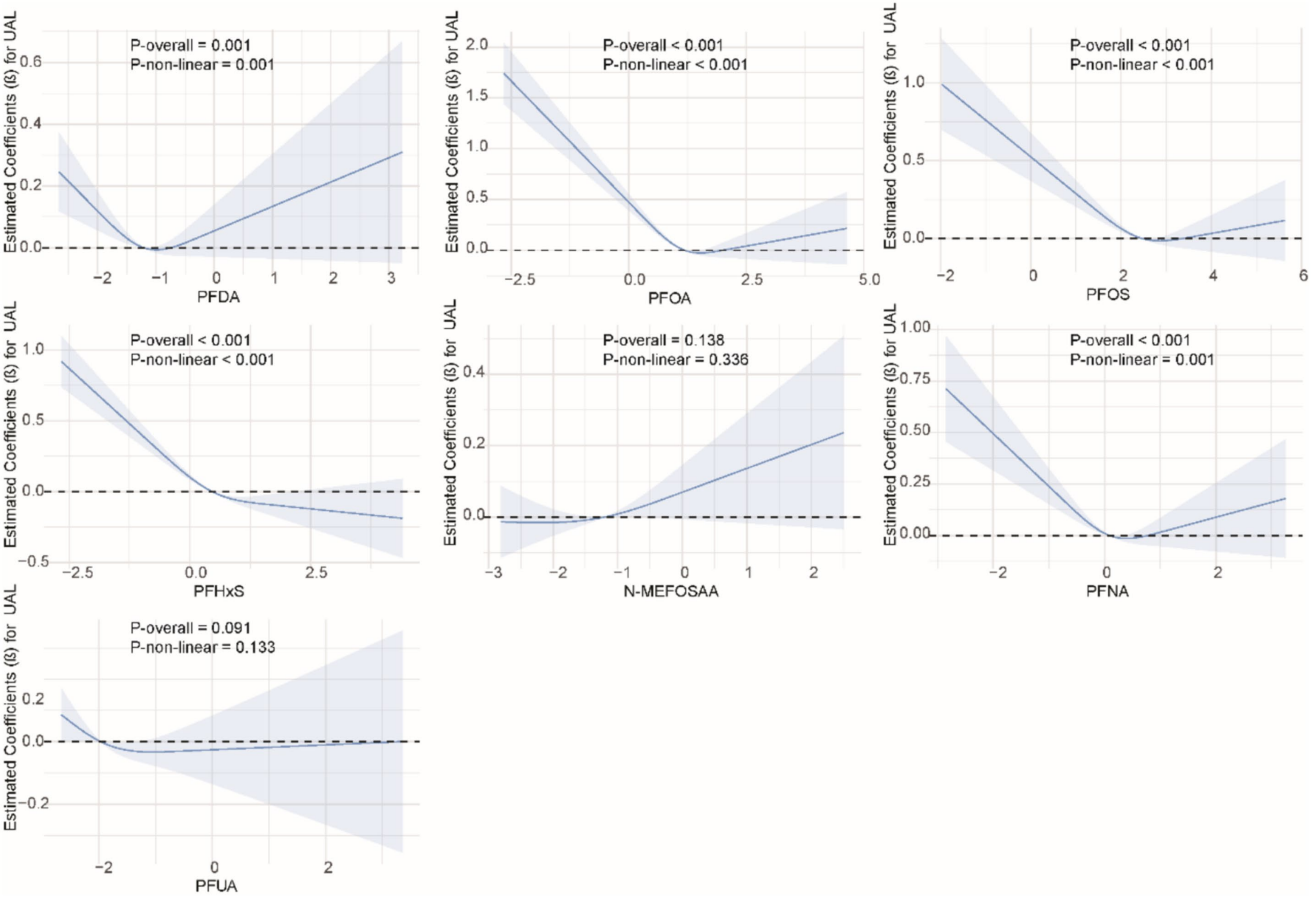
Non-linear dose–response relationships between individual PFASs and UAL.

**Figure 4:**
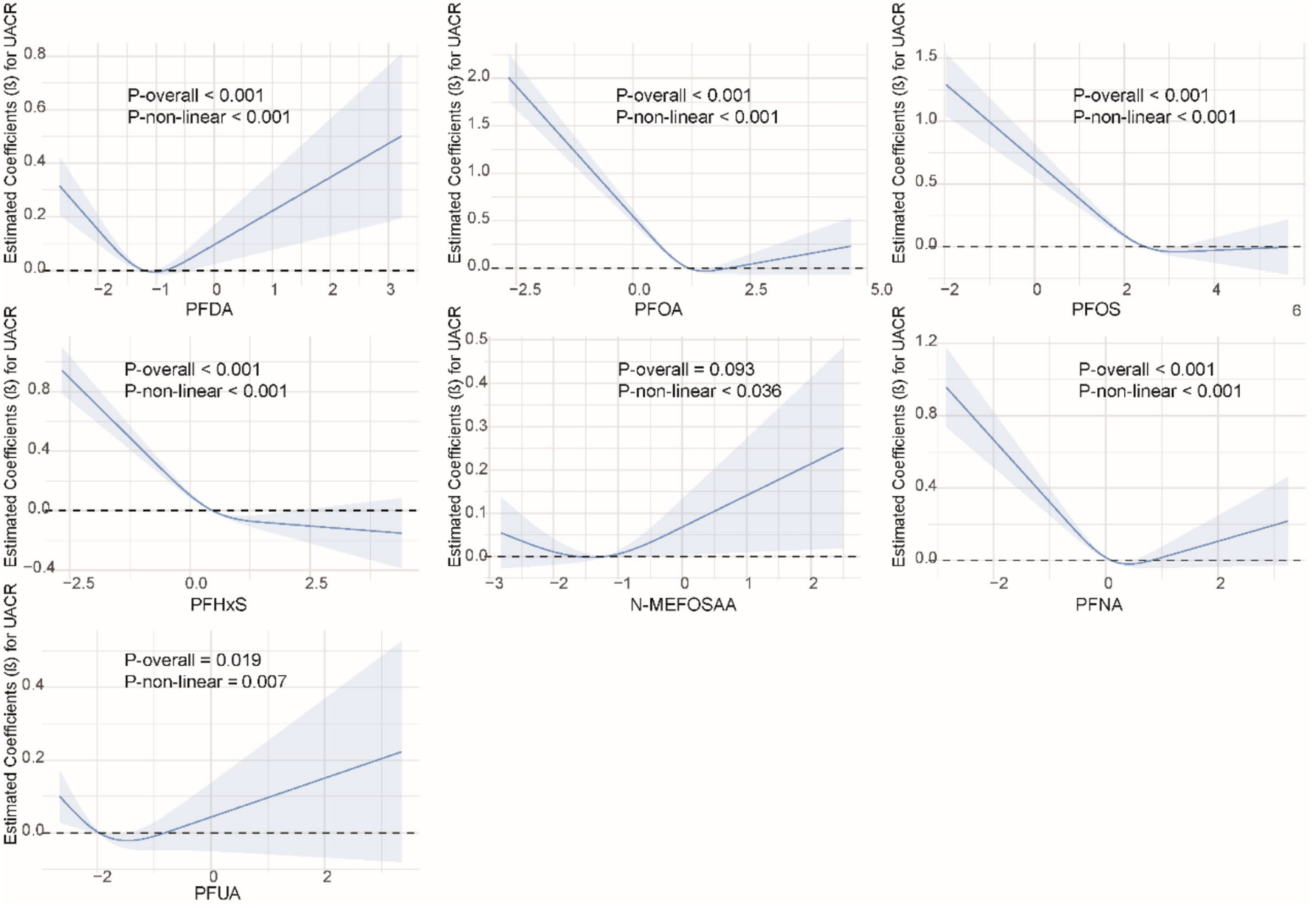
Non-linear dose–response relationships between individual PFASs and UACR.

We used restricted cubic splines to examine the non-linear relationship between PFASs and kidney function. As shown in Figure 1, we found significant nonlinear relationships between PFOA (*P*-overall <0.001, *P*-non-linear <0.001), PFOS (*P*-overall <0.001, *P*-non-linear = 0.002), PFHxS (*P*-overall = 0.001, *P*-non-linear = 0.020), PFNA (*P*-overall = 0.002, *P*-non-linear <0.001), and eGFR. The results of the nonlinear relationship between PFASs and UCR are presented in Figure 2. Among the PFASs, only PFNA exhibited a significant non-linear association with UCR (*P*-overall = 0.004, *P*-non-linear = 0.041). No significant nonlinear relationships were found between other PFASs and UCR (*P* > 0.05). The non-linear associations between PFAS and UAL were shown in Figure 3. Notably, PFDA (*P*-overall = 0.001, *P*-non-linear = 0.001), PFOA (*P*-overall <0.001, *P*-non-linear <0.001), PFOS (*P*-overall <0.001, *P*-non-linear <0.001), PFHxS (*P*-overall <0.001, *P*-non-linear <0.001), and PFNA (*P*-overall <0.001, *P*-non-linear = 0.001) were found to have significant non-linear associations with UAL. In Figure 4, we observe significant nonlinear relationships between all PFAS and UACR (*P*-non-linear <0.05). Except for N-MEFOSAA and PFUA, the overall *P*-values for other PFAS are less than 0.05.

### 3.5 Combined effect of PFASs on kidney function

**Figure 5:**
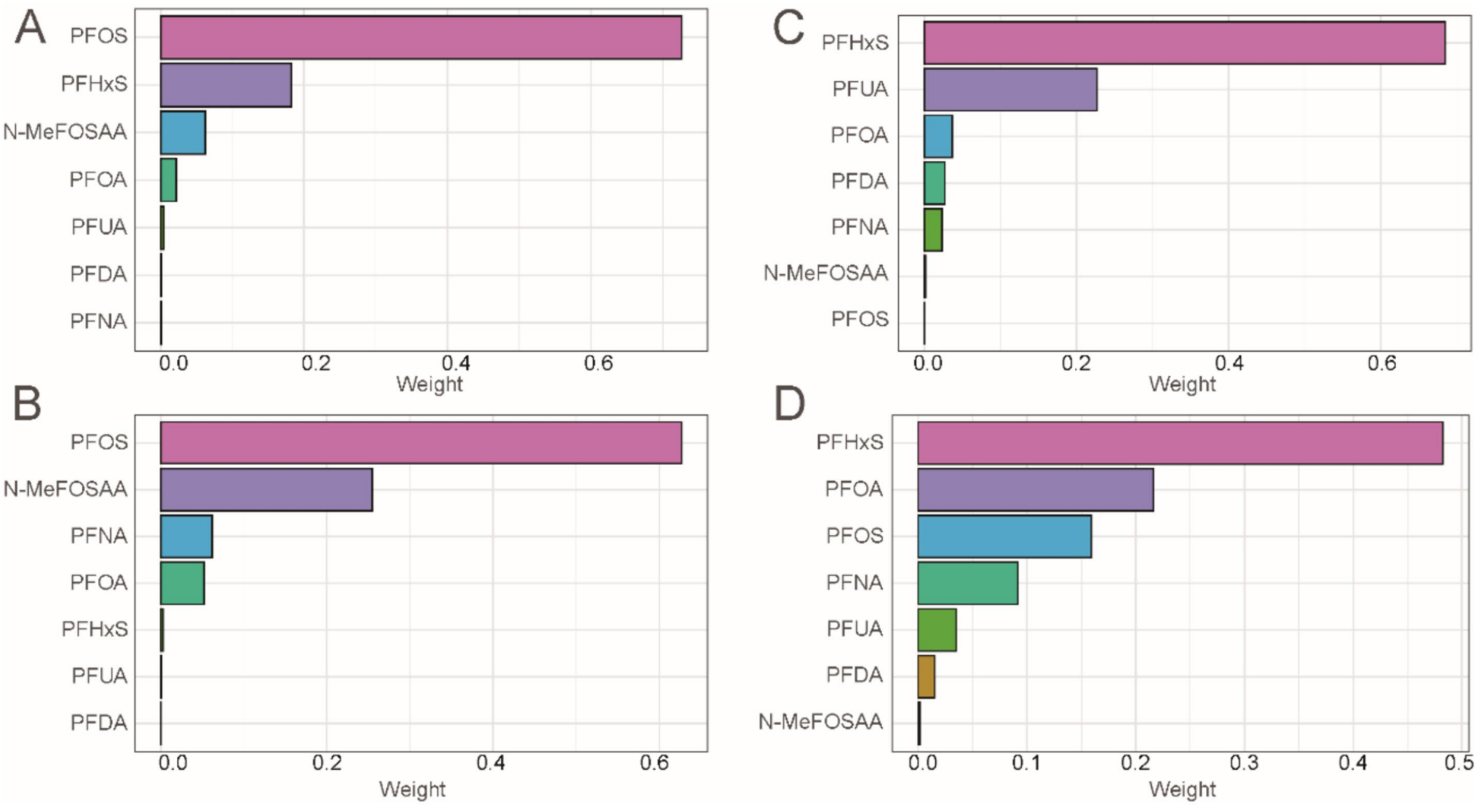
Combined effect of PFASs on kidney function in WQS regression. **(A)** WQS model regression index weights for eGFR. **(B)** WQS model regression index weights for UCR. **(C)** WQS model regression index weights for UAL. **(D)** WQS model regression index weights for UACR.

Considering the ubiquity of mixed exposure in the real world, we conducted a mixed exposure analysis. The results revealed significant associations between combined PFASs and kidney function. In the WQS model, mixed PFAS exposure was negatively correlated with eGFR, UAL and UACR (*β* = −0.49, 95% CI = −0.68, −0.29, *P* < 0.001; *β* = −0.21, 95% CI = −0.26, −0.15, *P* < 0.001; *β* = −0.19, 95% CI = −0.23, −0.15, *P* < 0.001), while the WQS index was positively correlated with UCR (*β* = 0.07, 95% CI = 0.04, 0.09, *P* < 0.001). The effects of mixed exposure were generally consistent with those of individual exposures. Detailed information is presented in Supplementary Table S3. We found that PFOS had a dominant weight in both eGFR and UCR (Figures 5A,B), while PFHxS had a dominant weight in UAL and UACR (Figures 5C,D).

**Figure 6:**
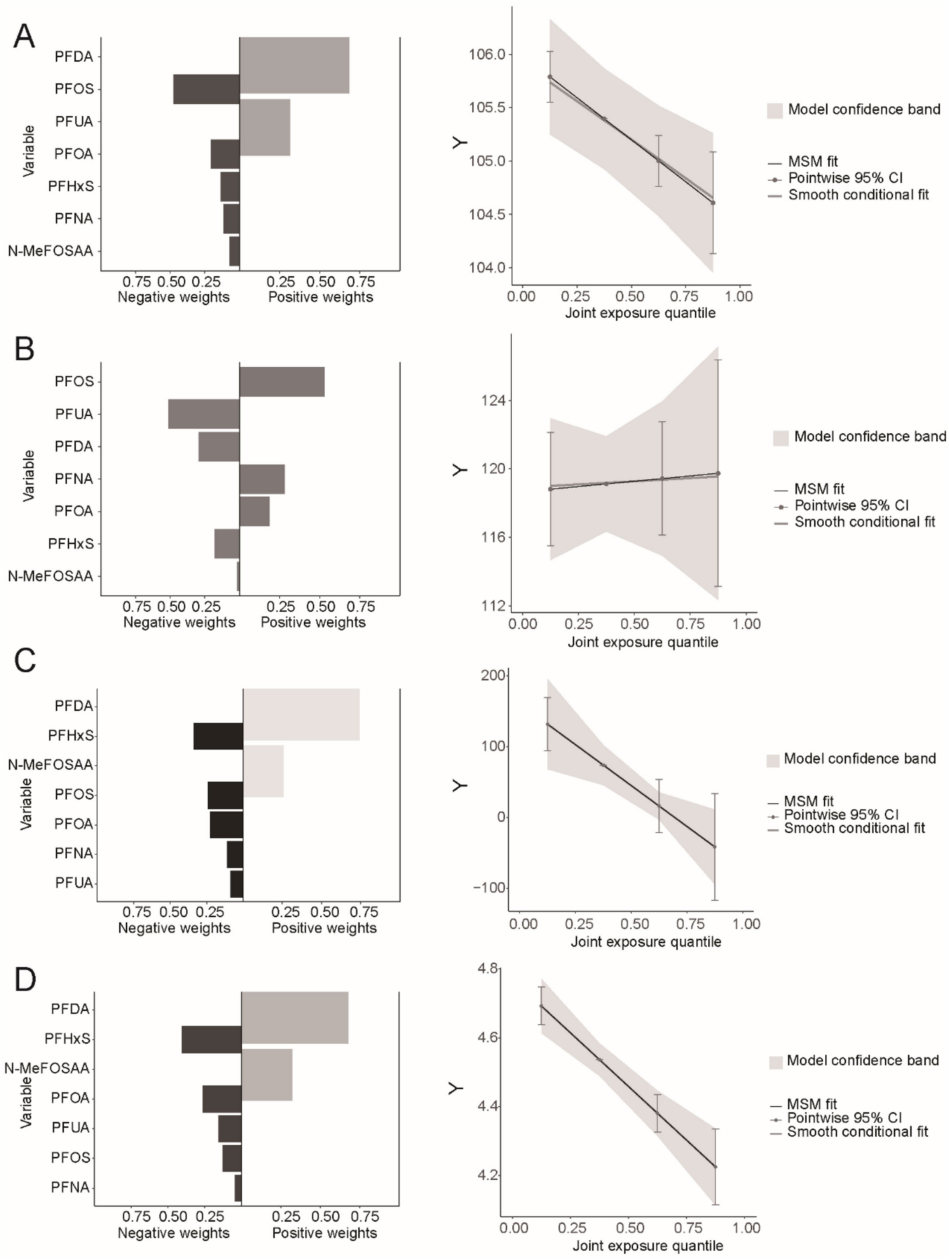
Combined effect of PFASs on kidney function in Q-gcomp regression. **(A)** The weight of bidirectional mixed effects and the linear relationship between PFAS mixed exposure and eGFR. **(B)** The weight of bidirectional mixed effects and the linear relationship between PFAS mixed exposure and UCR. **(C)** The weight of bidirectional mixed effects and the linear relationship between PFAS mixed exposure and UAL. **(D)** The weight of bidirectional mixed effects and the linear relationship between PFAS mixed exposure and UACR.

The results of the Q-gcomp analysis were present in Figure 6. In the negative effect of PFAS mixed exposure eGFR, UAL and UACR (Figures 6A,C,D) and the positive effect on UCR (Figure 5B). PFOS had the highest negative weight in eGFR while had the highest positive weight in UCR. PFHxS had the highest negative weight in UAL and UACR. Additionally, the association between mixed chemicals and eGFR, UAL and UACR showed a negative trend, while the association with UCR showed a positive trend. These results were consistent with those from the WQS model.

### 3.6 Subgroup analysis

Subgroup analysis (Supplementary Tables S4–S7) showed significant interactions between PFASs and various factors. For eGFR, PFDA was negatively correlated with eGFR in participants aged <55, drinkers, and other races, while PFOA was negatively correlated with eGFR in drinkers, those with BMI ≥25, and Non-Hispanic Whites. PFOS showed similar results in males, smokers, drinkers, and those with BMI ≥25. PFHxS was negatively correlated with eGFR in drinkers, those with BMI ≥25, Non-Hispanic Whites, and low education. N-MEFOSAA was negatively correlated with eGFR in participants aged ≥55, males, smokers, drinkers, and those with BMI ≥25 or low education. PFNA showed a negative correlation with eGFR in younger participants, drinkers, those with BMI ≥25, and other races. PFUA was negatively correlated with eGFR in participants aged <55 and positively correlated in non-smokers, non-drinkers, and other races.

For UCR, significant positive correlations were observed for PFOA in younger participants, males, and drinkers, and for PFOS in those with hypertension, drinkers, and BMI ≥25. PFHxS was positively correlated with UCR in participants with diabetes and other races, while N-MEFOSAA and PFNA showed positive correlations in participants of other races.

Age interacts with PFOA, PFHxS, PFNA, and UAL, with a significant negative correlation between PFOS, PFNA, and UAL in participants older than 55. Hypertension and diabetes interact with PFOA, PFOS, PFHxS, PFNA, and UAL, showing a significant negative correlation between PFOA, PFOS, PFNA, and UAL in the hypertensive group.

Regarding UACR, there are significant interactions between PFAS and variables such as gender, age, smoking status, hypertension, diabetes, and race. Detailed information is presented in Supplementary Table S7.

## 4 Discussion

In this national retrospective study, we reveal a connection between exposure to PFASs and two kidney function indicators, including eGFR, UCR, UAL and UACR. The results show a significant negative association between PFOA, PFOS, PFHxS, PFNA, and eGFR, UAL and UACR. Higher PFOA and PFOS concentrations in serum are significantly linked to increased UCR. Additionally, significant non-linear relationships were observed between PFAS and eGFR, UCR, UAL and UACR. In terms of mixed exposure, mixed PFASs show a significant negative association with eGFR, UAL and UACR, and a significant positive association with UCR. PFOS contributed the most in eGFR and UCR, while PFHxS contributed the most in UAL and UACR. Most subgroups show significant associations between PFAS exposure, eGFR, UCR, UAL and UACR. These results underscore the importance of considering multiple kidney function indicators when assessing the impact of PFAS exposure on renal health. By examining eGFR, UCR, UAL and UACR, our study provides a more comprehensive understanding of the potential nephrotoxic effects of PFAS. This approach enhances the robustness of our findings and highlights the need for multifaceted evaluations in future studies on PFAS-related kidney function alterations.

Previous studies have demonstrated that PFASs exposure can lead to kidney-related diseases. For example, in studies that directly measured exposure, PFAS exposure was significantly associated with decreased kidney function in healthy adolescents (24). Pharmacokinetic studies found that PFASs absorbed by the body distribute to serum, liver, kidneys, placenta, and umbilical cord serum. PFBA, PFDoA, and PFDA are highly concentrated in the kidneys, suggesting that the kidneys may be an important accumulation site for PFASs (25). In subjects living in Tarragona, the concentration of PFAS varied in different tissue samples, with PFBS, PFDoDA, and PFDA showing the highest concentrations in the kidneys, indicating that the kidneys may significantly accumulate PFASs (26). Besides accumulation, PFASs have a very slow metabolism in the body. Studies showed that the biological half-life of PFOA is 2.4 years, slightly longer in males than in females, and elimination is almost entirely through the kidneys (27). Another study from China found that the estimated renal clearance half-life of octafluorinated Cl-PFESA can reach 280 years (median), which is the highest biological persistence reported for PFAS in humans to date, demonstrating the high persistence of PFASs in the body (28). Toxicological studies indicate that male rats exposed to large amounts of PFASs exhibit acute toxicity, including changes in the glomeruli, peripheral edema, increased mortality, and chronic toxicity, which leads to weight loss and reduced kidney weight (29). In mice, PFOS exposure causes apoptosis in renal tubular cells, leading to epithelial-mesenchymal transition (EMT)-related kidney fibrosis and increased expression of EMT and kidney injury biomarkers, such as N-cadherin, vimentin, Snail, Kim1, and Lcn2 (30). *In vivo* experiments show increased apoptosis and higher levels of oxidative stress (through NFAT3, PPARγ, and SIRT1) (31). Another study found that exposure to PFBS, PFOA, PFOS, and PFNA reduces proliferation in African clawed frog A6 renal epithelial cells, leading to changes in DNA/RNA structure, protein structure, and fatty acid spectra (32). Recent studies have begun to focus on the potential molecular mechanisms of chronic kidney disease, particularly the impact of gut microbiota dysbiosis and metabolic changes on kidney health. Changes in the abundance of beneficial gut bacteria, such as Lactobacillus and Bifidobacterium, may significantly affect renal immune responses and inflammation (33, 34). Moreover, growing evidence suggests that gut microbiota may exacerbate kidney damage through lipid metabolism and tryptophan metabolism dysregulation (35–39). These metabolic disruptions not only affect energy metabolism but may also increase oxidative stress, leading to renal cell damage and fibrosis. In recent years, research has found that PFAS may influence gut microbiota, reducing beneficial bacteria levels and anti-inflammatory metabolites, thereby affecting kidney function (40, 41). Changes in the composition of gut microbiota and alterations in metabolites (such as fatty acids and amino acids) could also be key factors in PFAS-induced renal pathology, highlighting the need for further studies on how these metabolic pathways are altered after PFAS exposure and their impact on kidney function. Therefore, we hypothesize that PFAS may affect kidney health through multiple pathways, and these mechanisms may work synergistically, leading to chronic kidney injury. Future research should further explore the relationship between PFAS exposure and these mechanisms, as well as their specific roles in kidney damage.

Epidemiological studies also provide evidence about PFASs and kidney disease. High and extremely high levels of PFOA in the serum of fluorochemical workers in West Virginia, USA, may be associated with the occurrence of kidney cancer (42). In another cohort study, female serum PFOA levels were positively correlated with renal cell carcinoma, with a hazard ratio (HR) of 1.54 (95% CI: 1.05, 2.26) per doubling of PFOA, while this association was not found in males, indicating a gender difference (43). A health project in Shenyang, China, found that after adjusting for multiple confounding factors, most fluorinated PFAS, except PFOA and PFDA, were positively correlated with CKD (44). A cohort study in Tianjin, China, found significant correlations between PFOA and PFDA with uric acid, as well as PFOA with hyperuricemia in a single pollutant model in children’s serum (26).

In the research on perfluoroalkyl chemicals (PFASs) and kidney function, although some studies in recent years have preliminarily revealed an association between the two, research remains relatively limited, with most focusing on conventional kidney function indicators such as eGFR. For example, a study involving NHANES data showed that increased serum PFAS levels are associated with an elevated risk of CKD, and after adjusting for multiple covariates, PFOA and PFOS demonstrated a significant negative trend with eGFR (45). However, further adjustment for additional variables did not result in significant changes in eGFR, suggesting that while PFAS exposure is linked to kidney function decline, this association may be modulated by other factors. Our results are consistent with these findings, and in addition to PFOA and PFOS, our study also reveals a relationship between PFHxS, N-MEFOSAA, and eGFR, further expanding the research perspective on the impact of PFAS exposure on kidney function. Furthermore, certain groups exposed to higher concentrations of PFAS (such as children and adults living near PFAS manufacturing plants) also showed a trend of declining eGFR (46, 47). A cohort study in the U.S. found a negative correlation between serum PFOA levels and eGFR in self-reported postmenopausal women (48). Additionally, during a diabetes prevention program, participants’ serum PFAS concentrations were negatively correlated with eGFR (49). These findings not only support the association between PFAS exposure and kidney function decline but also suggest that susceptibility in specific populations may play a significant role in this process. However, we also observed that most existing literature focuses primarily on eGFR as a conventional indicator, overlooking other potential kidney function metrics. While eGFR is one of the standard indicators for assessing kidney health and has strong clinical applicability, it is not comprehensive. Especially in cases of long-term PFAS exposure, changes in eGFR may not fully reflect overall kidney dysfunction. Therefore, incorporating other kidney function indicators, particularly UCR,UAL and UACR, as supplementary assessments, could provide a more comprehensive evaluation. Our study corroborates this by demonstrating a significant negative correlation between PFAS exposure and both eGFR, UAL, and UACR, and further revealing a significant positive correlation between PFAS exposure and UCR. This finding provides another perspective on the potential disruptive effects of PFASs on kidney function, suggesting that evaluating kidney dysfunction through urinary biomarkers may be more sensitive and accurate than relying solely on eGFR in blood. From a deeper perspective, PFASs, as persistent organic pollutants, have a long half-life, allowing them to accumulate in the body and exert long-term effects on kidney detoxification and metabolic functions. The mechanisms by which PFAS impact kidney function via renal excretion may involve processes such as reabsorption of PFAS by renal tubules, changes in the activity of metabolic enzymes, and enhancement of renal inflammation (50–52). Therefore, future research should consider multidimensional indicators, combining both serum and urinary biomarkers, to comprehensively assess the potential risks of PFAS exposure on kidney health.

The impact of mixed PFAS exposure on kidney function is consistent with the findings of single-exposure studies. However, among various perfluoroalkyl compounds, PFOS has the most significant effect on eGFR and UCR. By comparing the distribution of serum PFOS levels in different study populations, we observed that, across all quartiles, the concentration of PFOS was consistently higher than that of other perfluoroalkyl compounds, including PFOA. These results suggest that the observed outcomes may be closely related to the distribution patterns of PFAS in different populations, with these distribution differences potentially being a key factor influencing kidney function. A kinetic study indicated that, after 24 h of oral exposure to various PFAS compounds, PFOS had the highest internal background in volunteers, suggesting that, compared to other PFAS, PFOS is less likely to be excreted through urine and instead accumulates more in the body (53). More importantly, the half-life of PFAS compounds in serum provides valuable insight into the mechanisms through which PFOS impacts kidney function. Studies have shown that PFAS compounds have long-term persistence in the body, with significant differences in their half-lives. For example, a study on Chinese chemical plant workers reported geometric mean half-lives for perfluorooctanoic acid and PFOS of 4.1 years and 4.0 years, respectively, while the half-life of perfluorooctane sulfonate was as long as 32.6 years (54). Another study on retired fluorochemical workers in the United States found the arithmetic mean half-life for PFOS to be 5.4 years, while that for PFOA was 3.8 years (55). These notably longer half-lives indicate that PFOS accumulates in the body for a significantly longer time compared to other PFAS compounds, potentially leading to more persistent and cumulative effects on kidney function. Over time, this long-term accumulation may exacerbate the damage PFOS causes to kidney function. Exposure of male rats to both PFOA and PFOS causes kidney hypertrophy and histopathological changes, including cell proliferation and damage, likely caused by reactive oxygen species (19, 56). Compared to PFOA, PFOS has a higher accumulation in the kidneys of rat models. We hypothesize that the slower excretion rate and longer half-life of PFOS contribute to its higher biological activity, which likely makes it the dominant compound in mixed exposures. Given the limited research comparing the renal toxicity of PFOS with other PFAS, we can only make preliminary hypotheses and conclusions. Further population-based studies and more in-depth mechanistic research are needed to confirm these findings.

Our research has several innovations. First, we not only investigated the impact of PFAS on kidney function but also considered multiple kidney function indicators, such as eGFR, UAL, UCR, and UACR, which distinguishes our approach from most existing studies that focus on a single kidney function marker. Through a comprehensive assessment of multiple indicators, we were able to provide a more thorough and precise depiction of the potential effects of PFAS exposure on kidney health. Second, previous studies have typically focused on the effects of a single PFAS chemical and overlooked the complexity of mixed exposure. Our study particularly emphasizes the impact of mixed PFAS exposure on renal function, providing us with new insights into the comprehensive effects of PFAS exposure from an innovative perspective. The results helped us find and focus on PFOS as a key chemical. Third, subgroup analyses help identify and understand the heterogeneity between groups, revealing different effects of PFAS exposure in various populations, which aids in determining specific groups for personalized preventive measures.

Our study also has some limitations. First, although a cross-sectional study is an effective tool, it is more suitable for descriptive research and preliminary exploration. Second, because data are collected at a single time point, it cannot capture dynamic changes over time, making it difficult to determine causal relationships between variables. Third, most variable information comes from retrospective questionnaire surveys, which may introduce information bias. Fourth, the selection of covariates may have issues of being too many or too few, leading to inaccurate model results. Fifth, the analytical results of epidemiological studies lack validation and require further confirmation through *in vivo* or *in vitro* experiments.

## 5 Conclusion

Our study revealed significant associations between PFAS exposure and various kidney function indicators. Specifically, exposures to PFOA, PFOS, PFHxS, and PFNA were negatively correlated with eGFR, UAL and UACR. Additionally, higher serum concentrations of PFOA and PFOS were significantly associated with increased UCR. Nonlinear relationships were found between PFAS and all kidney function indicators. Compared to single exposures, combined exposure to PFAS exhibited similar effects on kidney function. These findings provide an epidemiological perspective on how PFAS exposure may lead to kidney dysfunction, laying the groundwork for further research.

## Data Availability

Publicly available datasets were analyzed in this study. This data can be found at: https://wwwn.cdc.gov/nchs/nhanes/default.aspx.
