# Supplementary material for: Association between exposure to per- and polyfluoroalkyl substances and kidney function: a population study
Source: Front Med (Lausanne). 2025 Mar 26;12:1569031. doi: 10.3389/fmed.2025.1569031 (PMC11979136; doi:10.3389/fmed.2025.1569031)
Supplement: Supplementary file 1 [file Data_Sheet_1.pdf]

Supplementary material

Association between exposure to per- and polyfluoroalkyl substances (PFAS) and kidney function: A population study

Ning Huang [b 1], Yongping Cao [a 1], Xiaona Yang [c 1], Fei Ma [a], Hengyang Zhang [a], Wenwen Xiao [a *]

[a] Eastern Theater Command Centers for Disease Control and Prevention, Nanjing, China.

[b] Department of Urinary Surgery, Eastern Theater Naval Hospital of Chinese PLA, Zhoushan, Zhejiang, P. R. China.

[c]. Linping District Center for Disease Control and Prevention (Linping District Health Supervision Institute), Hangzhou, Zhejiang.

[1] Contributed equally.

Corresponding Author: Wenwen Xiao, Eastern Theater Command Centers for Disease Control and Prevention, 293 Zhongshan East Rd, Nanjing, China.

E-mail addresses: wenwenxiao1996@163.com.

Content

Table S1. Distribution of the selected PFAS.

Table S2. Multiple linear regression between individual PFAS and kidney function.

Table S3. WQS model regression between mixed PFAS and kidney function.

Table S4. Subgroup analysis for association between PFAS and eGFR.

Table S5. Subgroup analysis for association between PFAS and UCR.

Table S6. Subgroup analysis for association between PFAS and UAL.

Table S7. Subgroup analysis for association between PFAS and UACR.

Fig. S1. Flowchart of inclusion and exclusion.

Fig. S2. Spearman correlation of PFAS.

Table S1. Distribution of the selected PFAS.

Variables	Min	P5	P10	P25	P50	P75	P90	P95	Max	LOD (ng/mL)	>LOD (%)
PFDA	0.07	0.1	0.14	0.16	0.3	0.4	0.7	1.1	25.2	0.2	83.19
PFOA	0.07	0.9	1.3	2.1	3.27	5	7.144	8.93	104	0.1	99.68
PFOS	0.14	2.5	3.8	6.7	11.6	20	32.7	43.2	281	0.2	99.73
PFHxS	0.07	0.3	0.5	0.9	1.6	2.9	4.79	6.65	81.6	0.1	98.68
N-MEFOSAA	0.06	0.06	0.06	0.12	0.3	0.4	0.9	1.3	12.2	0.2	70.80
PFNA	0.06	0.4	0.5	0.74	1.1	1.64	2.6	3.4	25.75	0.1	99.43
PFUA	0.07	0.07	0.07	0.14	0.14	0.3	0.5	0.8	28.5	0.2	54.70

LOD, limit of detection.

Table S2. Multiple linear regression between individual PFAS and kidney function.

Outcomes	Model 1	P - value	Model 2	P - value
	**Estimate (95% CI)**		**Estimate (95% CI)**	
**eGFR**				
PFDA	-0.05 (-0.22, 0.13)	0.61	-0.07 (-0.25, 0.10)	0.41
PFOA	-0.24 (-0.43, -0.05)	0.01	-0.24 (-0.44, -0.05)	0.01
PFOS	-0.25 (-0.41, -0.09)	0.002	-0.27 (-0.43, -0.11)	<0.001
PFHxS	-0.22 (-0.36, -0.07)	0.003	-0.22 (-0.36, -0.08)	0.003
N-MEFOSAA	-0.19 (-0.33, -0.05)	0.008	-0.20 (-0.34, -0.06)	0.005
PFNA	-0.14 (-0.33, 0.06)	0.16	-0.14 (-0.33, 0.06)	0.17
PFUA	0.08 (-0.09, 0.26)	0.36	0.06 (-0.12, 0.23)	0.54
**UCR**				
PFDA	0.00 (-0.02, 0.02)	0.99	0.01 (-0.01, 0.03)	0.44
PFOA	0.06 (0.03, 0.09)	<0.001	0.06 (0.04, 0.09)	<0.001
PFOS	0.07 (0.04, 0.09)	<0.001	0.07 (0.05, 0.09)	<0.001
PFHxS	0.00 (-0.02, 0.02)	1	0.00 (-0.02, 0.02)	0.72
N-MEFOSAA	0.02 (0.00, 0.04)	0.04	0.03 (0.01, 0.05)	<0.001
PFNA	0.04 (0.02, 0.07)	0.002	0.04 (0.02, 0.07)	<0.001
PFUA	-0.03 (-0.05, -0.01)	0.02	-0.02 (-0.04, 0.01)	0.18
**UAL**				
PFDA	-0.08 (-0.12, -0.03)	0.001	-0.04 (-0.09, 0.00)	0.059
PFOA	-0.28 (-0.33, -0.23)	<0.001	-0.25 (-0.30, -0.20)	<0.001
PFOS	-0.14 (-0.18, -0.10)	<0.001	-0.12 (-0.16, -0.07)	<0.001
PFHxS	-0.21 (-0.24, -0.17)	<0.001	-0.19 (-0.23, -0.16)	<0.001
N-MEFOSAA	0.02 (-0.02, 0.06)	0.274	0.03 (-0.01, 0.07)	0.099
PFNA	-0.11 (-0.16, -0.07)	<0.001	-0.10 (-0.14, -0.05)	<0.001
PFUA	-0.07 (-0.11, -0.03)	0.002	-0.04 (-0.09, 0.01)	0.086
**UACR**				
PFDA	-0.06 (-0.10, -0.02)	0.004	-0.04 (-0.08, -0.01)	0.023
PFOA	-0.30 (-0.35, -0.26)	<0.001	-0.29 (-0.33, -0.24)	<0.001
PFOS	-0.18 (-0.21, -0.14)	<0.001	-0.17 (-0.20, -0.13)	<0.001
PFHxS	-0.20 (-0.23, -0.17)	<0.001	-0.19 (-0.22, -0.16)	<0.001
N-MEFOSAA	0.01 (-0.02, 0.04)	0.664	0.01 (-0.02, 0.04)	0.544
PFNA	-0.13 (-0.18, -0.09)	<0.001	-0.13 (-0.18, -0.09)	<0.001
PFUA	-0.03 (-0.07, 0.01)	0.002	-0.04 (-0.09, 0.01)	0.086

Model 1: adjusted for age, sex, race, education level and family income ratio. Model 2: adjusted

plus alcohol drinking, smoke status, diabetes, hypertension, body mass index category based on Model 1. CI, confidence interval.

Table S3. WQS model regression between mixed PFAS and kidney function.

**Outcomes**	**Estimate (95% CI)**	**P - value**
**eGFR**		
Positive	-0.13 (-0.38, 0.12)	0.32
Negative	-0.49 (-0.68, -0.29)	<0.001
**UCR**		
Positive	0.07 (0.04, 0.09)	<0.001
Negative	-0.03 (-0.07, 0)	0.05
**UAL**		
Positive	-0.03(-0.08, 0.02)	0.24
Negative	-0.21(-0.26, -0.15)	<0.001
**UACR**		
Positive	NA	NA
Negative	-0.19(-0.23,-0.15)	<0.001

The model was adjusted for age, sex, race, education level, family income ratio, alcohol drinking, smoke status, diabetes, hypertension, body mass index category. CI, confidence interval.

Table S4. Subgroup analysis for association between PFAS and eGFR.

Age	<55		≥55		
	Estimate (95% CI)	P - value	Estimate (95% CI)	P - value	P - int
PFDA	-0.57 (-1.04, -0.11)	0.02	0.25 (-0.14, 0.64)	0.2	0.01
PFOA	-0.38 (-0.89, 0.13)	0.15	0.31 (-0.15, 0.77)	0.18	0.03
PFOS	-1.20 (-1.63, -0.77)	<0.01	-0.43 (-0.80, -0.07)	0.02	0.03
PFHxS	-0.23 (-0.58, 0.12)	0.21	-0.08 (-0.44, 0.27)	0.64	0.46
N-MEFOSAA	-0.20 (-0.58, 0.18)	0.31	-0.88 (-1.19, -0.57)	<0.01	<0.01
PFNA	-0.94 (-1.44, -0.44)	<0.01	0.48 (0.05, 0.92)	0.03	<0.01
PFUA	-0.90 (-1.36, -0.45)	<0.01	0.04 (-0.35, 0.43)	0.83	0.005
Gender	Male		Female		
	Estimate (95% CI)	P - value	Estimate (95% CI)	P - value	P - int
PFDA	-0.09 (-0.32, 0.14)	0.43	0.02 (-0.24, 0.29)	0.86	0.78
PFOA	-0.06 (-0.32, 0.20)	0.67	-0.17 (-0.46, 0.11)	0.22	0.02
PFOS	-0.29 (-0.50, -0.08)	<0.01	-0.16 (-0.39, 0.07)	0.18	0.07
PFHxS	-0.04 (-0.24, 0.16)	0.67	-0.16 (-0.37, 0.04)	0.12	<0.01
N-MEFOSAA	-0.30 (-0.48, -0.11)	<0.01	-0.13 (-0.34, 0.08)	0.22	0.20
PFNA	-0.17 (-0.43, 0.08)	0.19	0.06 (-0.23, 0.35)	0.67	0.87
PFUA	-0.03 (-0.26, 0.19)	0.78	0.16 (-0.11, 0.44)	0.24	0.55
Smoke	Yes		No		
	Estimate (95% CI)	P - value	Estimate (95% CI)	P - value	P - int
PFDA	-0.17 (-0.42, 0.08)	0.18	0.03 (-0.22, 0.27)	0.83	0.62
PFOA	-0.13 (-0.40, 0.15)	0.35	-0.28 (-0.55, -0.02)	0.04	0.01
PFOS	-0.31 (-0.54, -0.09)	<0.01	-0.22 (-0.45, 0.00)	0.05	0.06
PFHxS	-0.14 (-0.35, 0.07)	0.18	-0.24 (-0.44, -0.05)	0.02	<0.01
N-MEFOSAA	-0.23 (-0.43, -0.03)	0.02	-0.19 (-0.38, 0.01)	0.06	0.30
PFNA	-0.17 (-0.45, 0.10)	0.21	-0.05 (-0.32, 0.23)	0.72	0.69
PFUA	-0.18 (-0.44, 0.08)	0.18	0.25 (0.01, 0.49)	0.05	0.10
Hypertension	Yes		No		
	Estimate (95% CI)	P - value	Estimate (95% CI)	P - value	P - int
PFDA	0.06 (-0.22, 0.34)	0.68	-0.08 (-0.30, 0.14)	0.48	0.95
PFOA	0.44 (0.14, 0.74)	<0.01	-0.60 (-0.84, -0.35)	<0.01	<0.01
PFOS	0.17 (-0.07, 0.42)	0.16	-0.51 (-0.71, -0.30)	<0.01	<0.01
PFHxS	0.34 (0.10, 0.58)	<0.01	-0.44 (-0.62, -0.27)	<0.01	<0.01
N-MEFOSAA	-0.15 (-0.37, 0.08)	0.20	-0.24 (-0.41, -0.06)	<0.01	0.37
PFNA	0.27 (-0.03, 0.57)	0.08	-0.32 (-0.57, -0.07)	0.01	<0.01
PFUA	0.07 (-0.22, 0.35)	0.65	0.08 (-0.15, 0.30)	0.50	0.32
Diabetes	Yes		No		
	Estimate (95% CI)	P - value	Estimate (95% CI)	P - value	P - int
PFDA	0.28 (-0.32, 0.89)	0.36	-0.08 (-0.26, 0.10)	0.39	0.78
PFOA	0.67 (0.06, 1.27)	0.03	-0.35 (-0.55, -0.15)	<0.01	<0.01
PFOS	0.28 (-0.25, 0.81)	0.30	-0.32 (-0.49, -0.15)	<0.01	0.05
PFHxS	0.49 (-0.03, 1.01)	0.06	-0.29 (-0.43, -0.14)	<0.01	<0.01
N-MEFOSAA	-0.19 (-0.65, 0.28)	0.43	-0.19 (-0.34, -0.05)	<0.01	0.95
PFNA	0.68 (0.00, 1.35)	0.05	-0.20 (-0.40, 0.00)	0.05	0.03

PFUA	-0.09 (-0.72, 0.53)	0.77	0.12 (-0.07, 0.30)	0.22	0.05
Alcohol	Yes		No		
	Estimate (95% CI)	P - value	Estimate (95% CI)	P - value	P - int
PFDA	-0.22 (-0.43, -0.02)	0.03	0.36 (0.02, 0.70)	0.04	0.04
PFOA	-0.32 (-0.55, -0.09)	<0.01	0.10 (-0.25, 0.45)	0.57	0.56
PFOS	-0.39 (-0.58, -0.21)	<0.01	0.10 (-0.20, 0.40)	0.52	0.76
PFHxS	-0.26 (-0.42, -0.09)	<0.01	-0.01 (-0.28, 0.27)	0.96	0.79
N-MEFOSAA	-0.20 (-0.37, -0.04)	0.01	-0.17 (-0.44, 0.10)	0.22	0.23
PFNA	-0.23 (-0.46, -0.01)	0.05	0.19 (-0.17, 0.55)	0.30	0.58
PFUA	-0.10 (-0.31, 0.10)	0.33	0.48 (0.14, 0.82)	<0.01	0.06
BMI	<25		≥25		
	Estimate (95% CI)	P - value	Estimate (95% CI)	P - value	P - int
PFDA	0.07 (-0.24, 0.39)	0.64	-0.13 (-0.34, 0.08)	0.23	0.13
PFOA	-0.06 (-0.41, 0.28)	0.71	-0.33 (-0.56, -0.10)	<0.01	0.04
PFOS	-0.15 (-0.44, 0.14)	0.32	-0.32 (-0.52, -0.13)	<0.01	0.03
PFHxS	-0.13 (-0.38, 0.12)	0.30	-0.26 (-0.44, -0.09)	<0.01	0.08
N-MEFOSAA	-0.23 (-0.48, 0.02)	0.07	-0.18 (-0.35, -0.01)	0.03	0.71
PFNA	0.09 (-0.26, 0.44)	0.62	-0.24 (-0.47, 0.00)	0.05	0.03
PFUA	0.12 (-0.19, 0.43)	0.45	0.03 (-0.18, 0.25)	0.76	0.34
Race	Non-Hispanic White		Other		
	Estimate (95% CI)	P - value	Estimate (95% CI)	P - value	P - int
PFDA	-0.16 (-0.38, 0.06)	0.15	1.25 (0.86, 1.64)	<0.01	<0.01
PFOA	-0.31 (-0.55, -0.07)	0.01	0.46 (0.04, 0.88)	0.03	0.06
PFOS	-0.39 (-0.59, -0.19)	<0.01	1.38 (1.04, 1.72)	<0.01	<0.01
PFHxS	-0.24 (-0.41, -0.06)	<0.01	0.55 (0.23, 0.87)	<0.01	0.01
N-MEFOSAA	-0.10 (-0.26, 0.07)	0.24	0.86 (0.55, 1.18)	<0.01	<0.01
PFNA	-0.14 (-0.37, 0.09)	0.22	1.17 (0.72, 1.61)	<0.01	<0.01
PFUA	-0.13 (-0.36, 0.10)	0.25	1.20 (0.84, 1.57)	<0.01	<0.01
Education	Less than high school		Other		
	Estimate (95% CI)	P - value	Estimate (95% CI)	P - value	P - int
PFDA	0.02 (-0.24, 0.28)	0.89	-0.11 (-0.35, 0.13)	0.38	0.98
PFOA	-0.26 (-0.54, 0.03)	0.08	-0.21 (-0.47, 0.04)	0.10	0.45
PFOS	-0.28 (-0.51, -0.04)	0.02	-0.22 (-0.44, -0.01)	0.04	0.22
PFHxS	-0.16 (-0.37, 0.06)	0.15	-0.26 (-0.45, -0.07)	<0.01	0.93
N-MEFOSAA	-0.26 (-0.47, -0.05)	0.01	-0.14 (-0.32, 0.05)	0.15	0.13
PFNA	-0.11 (-0.39, 0.18)	0.45	-0.12 (-0.39, 0.15)	0.38	0.46
PFUA	0.20 (-0.06, 0.46)	0.14	-0.06 (-0.30, 0.18)	0.62	0.71

The model was adjusted for age, sex, race, education level, family income ratio, alcohol drinking, smoke status, diabetes, hypertension, body mass index category. CI, confidence interval.

Table S5. Subgroup analysis for association between PFAS and UCR.

Age	<55		≥55		
	Estimate (95% CI)	P - value	Estimate (95% CI)	P - value	P - int
PFDA	-2.71 (-6.36, 0.95)	0.15	0.21 (-3.77, 4.19)	0.92	0.41
PFOA	1.33 (0.41, 2.24)	<0.01	0.70 (-0.22, 1.62)	0.14	0.23
PFOS	0.22 (0.01, 0.43)	0.04	0.28 (0.14, 0.41)	<0.01	0.93
PFHxS	0.66 (-0.20, 1.52)	0.13	-0.15 (-1.13, 0.83)	0.76	0.14
N-MEFOSAA	3.38 (-1.92, 8.69)	0.21	-0.10 (-4.44, 4.23)	0.96	0.30
PFNA	1.11 (-1.17, 3.40)	0.34	0.75 (-1.11, 2.62)	0.43	0.64
PFUA	-3.15 (-6.94, 0.65)	0.10	1.62 (-4.68, 7.93)	0.61	0.36
Gender	Male		Female		
	Estimate (95% CI)	P - value	Estimate (95% CI)	P - value	P - int
PFDA	-1.25 (-4.38, 1.88)	0.43	-2.22 (-8.45, 4.01)	0.49	0.87
PFOA	1.13 (0.29, 1.97)	0.01	0.85 (-0.25, 1.94)	0.13	0.62
PFOS	0.31 (0.16, 0.46)	<0.01	0.24 (0.02, 0.46)	0.03	0.75
PFHxS	0.68 (-0.12, 1.47)	0.09	-0.59 (-1.78, 0.60)	0.33	0.10
N-MEFOSAA	2.05 (-3.00, 7.10)	0.43	2.65 (-2.11, 7.42)	0.28	0.68
PFNA	1.04 (-1.19, 3.26)	0.36	1.03 (-0.97, 3.03)	0.31	0.90
PFUA	-2.34 (-6.18, 1.51)	0.23	0.23 (-5.81, 6.28)	0.94	0.47
Smoke	Yes		No		
	Estimate (95% CI)	P - value	Estimate (95% CI)	P - value	P - int
PFDA	-3.36 (-7.40, 0.69)	0.10	0.13 (-3.54, 3.79)	0.95	0.28
PFOA	1.18 (0.15, 2.20)	0.02	1.00 (0.16, 1.85)	0.02	0.66
PFOS	0.22 (0.05, 0.39)	0.01	0.34 (0.16, 0.51)	<0.01	0.19
PFHxS	0.10 (-0.93, 1.12)	0.86	0.59 (-0.24, 1.41)	0.16	0.18
N-MEFOSAA	1.84 (-3.13, 6.80)	0.47	2.88 (-2.00, 7.76)	0.25	0.57
PFNA	0.30 (-1.91, 2.51)	0.79	1.61 (-0.42, 3.64)	0.12	0.36
PFUA	-3.01 (-7.38, 1.35)	0.18	-0.51 (-5.18, 4.16)	0.83	0.62
Hypertension	Yes		No		
	Estimate (95% CI)	P - value	Estimate (95% CI)	P - value	P - int
PFDA	3.00 (-3.43, 9.43)	0.36	-2.10 (-5.13, 0.93)	0.18	0.28
PFOA	1.32 (0.21, 2.42)	0.02	0.98 (0.17, 1.79)	0.02	0.95
PFOS	0.48 (0.31, 0.65)	<0.01	0.14 (-0.03, 0.30)	0.11	0.05
PFHxS	0.83 (-0.31, 1.97)	0.15	0.22 (-0.57, 1.00)	0.59	0.67
N-MEFOSAA	-0.81 (-6.05, 4.43)	0.76	4.20 (-0.39, 8.79)	0.07	0.10
PFNA	2.01 (-0.12, 4.15)	0.06	0.44 (-1.63, 2.50)	0.68	0.59
PFUA	2.27 (-4.23, 8.76)	0.49	-2.60 (-6.28, 1.07)	0.16	0.30
Diabetes	Yes		No		
	Estimate (95% CI)	P - value	Estimate (95% CI)	P - value	P - int
PFDA	-3.90 (-15.33, 7.53)	0.50	-1.25 (-4.06, 1.56)	0.38	0.71
PFOA	1.93 (0.19, 3.67)	0.03	0.92 (0.22, 1.63)	0.01	0.14
PFOS	0.52 (0.26, 0.77)	<0.01	0.21 (0.08, 0.35)	<0.01	0.03
PFHxS	3.42 (1.10, 5.74)	<0.01	0.13 (-0.54, 0.80)	0.71	<0.01
N-MEFOSAA	0.20 (-9.02, 9.42)	0.97	2.36 (-1.38, 6.09)	0.22	0.91
PFNA	1.25 (-2.38, 4.89)	0.50	0.97 (-0.66, 2.60)	0.24	0.76
PFUA	-3.88 (-16.96, 9.20)	0.56	-1.64 (-4.94, 1.65)	0.33	0.83
Alcohol	Yes		No		
	Estimate (95% CI)	P - value	Estimate (95% CI)	P - value	P - int

PFDA	-2.10 (-5.64, 1.45)	0.25	-0.47 (-4.59, 3.65)	0.82	0.68
PFOA	1.57 (0.68, 2.46)	<0.01	0.48 (-0.45, 1.41)	0.31	0.16
PFOS	0.34 (0.18, 0.49)	<0.01	0.17 (-0.03, 0.37)	0.09	0.40
PFHxS	0.42 (-0.33, 1.16)	0.27	0.23 (-1.04, 1.51)	0.72	0.97
N-MEFOSAA	1.96 (-2.57, 6.49)	0.40	2.34 (-2.96, 7.63)	0.39	0.70
PFNA	1.19 (-0.65, 3.03)	0.21	0.68 (-1.85, 3.21)	0.60	0.63
PFUA	-2.87 (-6.64, 0.90)	0.14	1.34 (-4.54, 7.22)	0.65	0.42
BMI	<25		≥25		
	Estimate (95% CI)	P - value	Estimate (95% CI)	P - value	P - int
PFDA	-2.70 (-6.76, 1.36)	0.19	-0.20 (-3.87, 3.46)	0.91	0.26
PFOA	1.38 (0.06, 2.70)	0.04	0.93 (0.17, 1.69)	0.02	0.49
PFOS	0.16 (-0.06, 0.38)	0.15	0.33 (0.18, 0.48)	<0.01	0.37
PFHxS	0.11 (-1.14, 1.36)	0.87	0.35 (-0.40, 1.11)	0.36	0.83
N-MEFOSAA	6.47 (0.75, 12.18)	0.03	-0.94 (-5.33, 3.45)	0.68	0.02
PFNA	1.08 (-2.00, 4.15)	0.49	1.07 (-0.65, 2.79)	0.22	0.97
PFUA	-2.88 (-7.01, 1.25)	0.17	0.58 (-4.44, 5.59)	0.82	0.20
Race	Non-Hispanic White		Other		
	Estimate (95% CI)	P - value	Estimate (95% CI)	P - value	P - int
PFDA	0.31 (-6.90, 7.52)	0.93	-1.03 (-4.11, 2.06)	0.51	0.65
PFOA	0.91 (0.01, 1.81)	0.05	1.75 (0.78, 2.72)	<0.01	0.22
PFOS	0.30 (0.11, 0.49)	<0.01	0.44 (0.27, 0.60)	<0.01	0.47
PFHxS	-0.29 (-1.11, 0.52)	0.48	1.76 (0.72, 2.80)	<0.01	<0.01
N-MEFOSAA	0.03 (-4.28, 4.34)	0.99	10.23 (4.50, 15.95)	<0.01	0.01
PFNA	1.09 (-0.94, 3.12)	0.29	2.59 (0.38, 4.81)	0.02	0.35
PFUA	3.93 (-2.70, 10.55)	0.25	-2.63 (-6.40, 1.14)	0.17	0.07
Education	Less than high school		Other		
	Estimate (95% CI)	P - value	Estimate (95% CI)	P - value	P - int
PFDA	-1.91 (-5.31, 1.48)	0.27	0.08 (-4.43, 4.58)	0.97	0.33
PFOA	0.80 (0.00, 1.59)	0.05	1.79 (0.67, 2.91)	<0.01	0.15
PFOS	0.18 (0.01, 0.34)	0.04	0.43 (0.25, 0.61)	<0.01	0.01
PFHxS	0.12 (-0.82, 1.05)	0.81	0.68 (-0.21, 1.57)	0.13	0.33
N-MEFOSAA	2.36 (-1.80, 6.51)	0.27	1.99 (-4.15, 8.14)	0.53	0.93
PFNA	0.65 (-1.14, 2.44)	0.48	2.18 (-0.46, 4.82)	0.11	0.25
PFUA	-1.23 (-4.72, 2.26)	0.49	-3.18 (-10.55, 4.20)	0.40	0.85

The model was adjusted for age, sex, race, education level, family income ratio, alcohol drinking, smoke status, diabetes, hypertension, body mass index category. CI, confidence interval.

Table S6. Subgroup analysis for association between PFAS and UAL.

Age	<55		≥55		
	Estimate (95% CI)	P - value	Estimate (95% CI)	P - value	P - int
PFDA	-0.03 (-0.09, 0.04)	0.710	-0.03 (-0.09, 0.04)	0.044	0.117
PFOA	-0.11 (-0.18, -0.04)	<0.001	-0.11 (-0.18, -0.04)	<0.001	<0.001
PFOS	-0.04 (-0.10, 0.02)	0.066	-0.04 (-0.10, 0.02)	<0.001	0.429
PFHxS	-0.13 (-0.18, -0.08)	<0.001	-0.13 (-0.18, -0.08)	<0.001	0.004
N-MEFOSAA	0.06 (0.01, 0.11)	0.266	0.06 (0.01, 0.11)	0.096	0.102
PFNA	-0.03 (-0.10, 0.04)	0.534	-0.03 (-0.10, 0.04)	0.001	0.020
PFUA	-0.05 (-0.11, 0.02)	0.761	-0.05 (-0.11, 0.02)	0.223	0.420
Gender	Male		Female		
	Estimate (95% CI)	P - value	Estimate (95% CI)	P - value	P - int
PFDA	-0.06 (-0.12, 0.01)	0.078	-0.01 (-0.07, 0.06)	0.857	0.380
PFOA	-0.22 (-0.29, -0.15)	<0.001	-0.18 (-0.24, -0.11)	<0.001	0.835
PFOS	-0.09 (-0.15, -0.04)	0.002	-0.08 (-0.14, -0.03)	0.004	0.305
PFHxS	-0.19 (-0.24, -0.14)	<0.001	-0.15 (-0.20, -0.10)	<0.001	0.587
N-MEFOSAA	0.04 (-0.01, 0.09)	0.097	0.03 (-0.02, 0.08)	0.268	0.268
PFNA	-0.09 (-0.16, -0.02)	0.017	-0.07 (-0.14, 0.00)	0.047	0.543
PFUA	-0.06 (-0.12, 0.01)	0.077	-0.01 (-0.07, 0.06)	0.800	0.553
Smoke	Yes		No		
	Estimate (95% CI)	P - value	Estimate (95% CI)	P - value	P - int
PFDA	-0.07 (-0.14, 0.00)	0.043	0.00 (-0.06, 0.06)	0.909	0.338
PFOA	-0.25 (-0.33, -0.18)	<0.001	-0.20 (-0.26, -0.13)	<0.001	0.419
PFOS	-0.11 (-0.17, -0.05)	0.001	-0.09 (-0.14, -0.04)	0.001	0.474
PFHxS	-0.22 (-0.27, -0.16)	<0.001	-0.15 (-0.20, -0.11)	<0.001	0.257
N-MEFOSAA	0.06 (0.01, 0.12)	0.023	0.02 (-0.03, 0.06)	0.459	0.057
PFNA	-0.09 (-0.17, -0.02)	0.015	-0.09 (-0.15, -0.02)	0.012	0.647
PFUA	-0.05 (-0.12, 0.02)	0.194	-0.02 (-0.08, 0.04)	0.503	0.894
Hypertension	Yes		No		
	Estimate (95% CI)	P - value	Estimate (95% CI)	P - value	P - int
PFDA	-0.08 (-0.17, 0.01)	0.066	0.00 (-0.05, 0.05)	0.971	0.235
PFOA	-0.48 (-0.57, -0.39)	<0.001	-0.05 (-0.10, 0.01)	0.105	<0.001
PFOS	-0.25 (-0.32, -0.17)	<0.001	0.00 (-0.04, 0.05)	0.932	<0.001
PFHxS	-0.33 (-0.41, -0.26)	<0.001	-0.11 (-0.15, -0.07)	<0.001	<0.001
N-MEFOSAA	-0.02 (-0.09, 0.05)	0.545	0.06 (0.03, 0.10)	0.001	0.058
PFNA	-0.19 (-0.29, -0.10)	<0.001	-0.02 (-0.07, 0.04)	0.599	0.004
PFUA	-0.01 (-0.10, 0.08)	0.845	-0.05 (-0.10, 0.01)	0.081	0.204
Diabetes	Yes		No		
	Estimate (95% CI)	P - value	Estimate (95% CI)	P - value	P - int
PFDA	-0.07 (-0.25, 0.12)	0.488	-0.03 (-0.08, 0.01)	0.160	0.619
PFOA	-0.78 (-0.95, -0.60)	<0.001	-0.13 (-0.18, -0.08)	<0.001	<0.001
PFOS	-0.40 (-0.55, -0.24)	<0.001	-0.05 (-0.10, -0.01)	0.008	<0.001
PFHxS	-0.55 (-0.70, -0.40)	<0.001	-0.14 (-0.17, -0.10)	<0.001	<0.001
N-MEFOSAA	-0.01 (-0.15, 0.14)	0.939	0.04 (0.01, 0.08)	0.015	0.302
PFNA	-0.40 (-0.60, -0.20)	<0.001	-0.05 (-0.10, 0.00)	0.046	<0.001
PFUA	0.03 (-0.16, 0.22)	0.749	-0.04 (-0.09, 0.00)	0.078	0.490
Alcohol	Yes		No		

	Estimate (95% CI)	P - value	Estimate (95% CI)	P - value	P - int
PFDA	-0.04 (-0.09, 0.02)	0.183	-0.04 (-0.13, 0.05)	0.346	0.984
PFOA	-0.20 (-0.26, -0.15)	<0.001	-0.27 (-0.36, -0.18)	<0.001	0.220
PFOS	-0.10 (-0.15, -0.05)	<0.001	-0.10 (-0.18, -0.02)	0.011	0.838
PFHxS	-0.19 (-0.24, -0.15)	<0.001	-0.17 (-0.24, -0.10)	<0.001	0.355
N-MEFOSAA	0.03 (-0.01, 0.07)	0.210	0.06 (-0.01, 0.13)	0.092	0.432
PFNA	-0.06 (-0.12, 0.00)	0.044	-0.16 (-0.26, -0.07)	0.001	0.066
PFUA	-0.04 (-0.09, 0.01)	0.156	-0.03 (-0.11, 0.06)	0.570	0.651
BMI	<25		≥25		
	Estimate (95% CI)	P - value	Estimate (95% CI)	P - value	P - int
PFDA	0.00 (-0.09, 0.08)	0.953	-0.06 (-0.11, 0.00)	0.040	0.489
PFOA	-0.18 (-0.27, -0.09)	<0.001	-0.24 (-0.30, -0.19)	<0.001	0.432
PFOS	-0.06 (-0.14, 0.01)	0.113	-0.12 (-0.17, -0.07)	<0.001	0.445
PFHxS	-0.15 (-0.22, -0.09)	<0.001	-0.21 (-0.25, -0.16)	<0.001	0.381
N-MEFOSAA	0.03 (-0.04, 0.10)	0.365	0.03 (-0.01, 0.07)	0.161	0.850
PFNA	-0.04 (-0.14, 0.05)	0.346	-0.11 (-0.17, -0.05)	<0.001	0.431
PFUA	-0.08 (-0.16, 0.00)	0.049	-0.02 (-0.07, 0.04)	0.479	0.120
Race	Non-Hispanic White		Other		
	Estimate (95% CI)	P - value	Estimate (95% CI)	P - value	P - int
PFDA	-0.03 (-0.09, 0.04)	0.424	-0.03 (-0.09, 0.03)	0.374	0.669
PFOA	-0.11 (-0.18, -0.04)	0.003	-0.31 (-0.37, -0.24)	<0.001	<0.001
PFOS	-0.04 (-0.10, 0.02)	0.196	-0.12 (-0.18, -0.07)	<0.001	0.011
PFHxS	-0.13 (-0.18, -0.08)	<0.001	-0.23 (-0.28, -0.18)	<0.001	0.005
N-MEFOSAA	0.06 (0.01, 0.11)	0.018	0.02 (-0.03, 0.08)	0.356	0.226
PFNA	-0.03 (-0.10, 0.04)	0.352	-0.13 (-0.20, -0.06)	<0.001	0.028
PFUA	-0.05 (-0.11, 0.02)	0.182	-0.01 (-0.07, 0.05)	0.670	0.786
Education	Less than high school		Other		
	Estimate (95% CI)	P - value	Estimate (95% CI)	P - value	P - int
PFDA	-0.05 (-0.11, 0.02)	0.141	-0.02 (-0.08, 0.05)	0.634	0.516
PFOA	-0.23 (-0.30, -0.16)	<0.001	-0.21 (-0.28, -0.14)	<0.001	0.513
PFOS	-0.11 (-0.17, -0.05)	<0.001	-0.08 (-0.14, -0.03)	0.004	0.538
PFHxS	-0.20 (-0.25, -0.14)	<0.001	-0.17 (-0.22, -0.12)	<0.001	0.641
N-MEFOSAA	0.06 (0.01, 0.11)	0.023	0.02 (-0.03, 0.07)	0.499	0.080
PFNA	-0.10 (-0.17, -0.03)	0.005	-0.07 (-0.14, 0.00)	0.053	0.915
PFUA	-0.03 (-0.10, 0.04)	0.376	-0.03 (-0.10, 0.03)	0.290	0.990

Table S7. Subgroup analysis for association between PFAS and UACR.

Age	<55		≥55		
	Estimate (95% CI)	P - value	Estimate (95% CI)	P - value	P - int
PFDA	0.00 (-0.04, 0.05)	0.936	-0.09 (-0.16, -0.02)	0.011	0.067
PFOA	-0.19 (-0.24, -0.14)	<0.001	-0.40 (-0.48, -0.32)	<0.001	<0.001
PFOS	-0.12 (-0.16, -0.08)	<0.001	-0.20 (-0.27, -0.14)	<0.001	0.324
PFHxS	-0.13 (-0.16, -0.09)	<0.001	-0.27 (-0.34, -0.21)	<0.001	0.001
N-MEFOSAA	-0.01 (-0.04, 0.03)	0.729	0.03 (-0.02, 0.09)	0.226	0.026
PFNA	-0.07 (-0.12, -0.02)	0.006	-0.19 (-0.26, -0.11)	<0.001	0.016
PFUA	0.02 (-0.02, 0.07)	0.333	-0.04 (-0.11, 0.03)	0.212	0.113
Gender	Male		Female		
	Estimate (95% CI)	P - value	Estimate (95% CI)	P - value	P - int
PFDA	-0.06 (-0.12, -0.01)	0.024	-0.02 (-0.07, 0.04)	0.542	0.263
PFOA	-0.30 (-0.36, -0.24)	<0.001	-0.24 (-0.29, -0.18)	<0.001	0.220
PFOS	-0.17 (-0.22, -0.12)	<0.001	-0.15 (-0.20, -0.10)	<0.001	0.618
PFHxS	-0.23 (-0.27, -0.18)	<0.001	-0.13 (-0.17, -0.09)	<0.001	0.008
N-MEFOSAA	0.01 (-0.04, 0.05)	0.692	0.00 (-0.04, 0.04)	0.916	0.317
PFNA	-0.13 (-0.20, -0.07)	<0.001	-0.11 (-0.17, -0.06)	<0.001	0.800
PFUA	-0.04 (-0.09, 0.02)	0.169	0.01 (-0.04, 0.06)	0.728	0.380
Smoke	Yes		No		
	Estimate (95% CI)	P - value	Estimate (95% CI)	P - value	P - int
PFDA	-0.07 (-0.13, -0.01)	0.017	-0.02 (-0.07, 0.03)	0.497	0.391
PFOA	-0.31 (-0.38, -0.25)	<0.001	-0.25 (-0.31, -0.20)	<0.001	0.650
PFOS	-0.16 (-0.22, -0.11)	<0.001	-0.17 (-0.22, -0.13)	<0.001	0.052
PFHxS	-0.22 (-0.26, -0.17)	<0.001	-0.16 (-0.20, -0.12)	<0.001	0.717
N-MEFOSAA	0.04 (-0.01, 0.08)	0.140	-0.01 (-0.05, 0.03)	0.542	0.009
PFNA	-0.14 (-0.20, -0.07)	<0.001	-0.13 (-0.18, -0.07)	<0.001	0.554
PFUA	-0.03 (-0.09, 0.03)	0.381	0.00 (-0.05, 0.05)	0.898	0.933
Hypertension	Yes		No		
	Estimate (95% CI)	P - value	Estimate (95% CI)	P - value	P - int
PFDA	-0.12 (-0.21, -0.04)	0.003	0.00 (-0.03, 0.04)	0.808	0.017
PFOA	-0.55 (-0.63, -0.47)	<0.001	-0.11 (-0.15, -0.06)	<0.001	<0.001
PFOS	-0.34 (-0.41, -0.27)	<0.001	-0.05 (-0.09, -0.02)	0.004	<0.001
PFHxS	-0.37 (-0.44, -0.30)	<0.001	-0.09 (-0.13, -0.06)	<0.001	<0.001
N-MEFOSAA	-0.04 (-0.10, 0.03)	0.269	0.03 (0.00, 0.06)	0.052	0.141
PFNA	-0.27 (-0.36, -0.19)	<0.001	-0.04 (-0.08, 0.00)	0.078	0.000
PFUA	-0.03 (-0.11, 0.05)	0.476	-0.01 (-0.05, 0.03)	0.682	0.948
Diabetes	Yes		No		
	Estimate (95% CI)	P - value	Estimate (95% CI)	P - value	P - int
PFDA	-0.06 (-0.24, 0.12)	0.497	-0.04 (-0.08, -0.01)	0.020	0.699
PFOA	-0.83 (-1.00, -0.67)	<0.001	-0.19 (-0.23, -0.15)	<0.001	<0.001
PFOS	-0.46 (-0.61, -0.31)	<0.001	-0.12 (-0.16, -0.09)	<0.001	<0.001
PFHxS	-0.60 (-0.75, -0.46)	<0.001	-0.13 (-0.16, -0.10)	<0.001	<0.001
N-MEFOSAA	-0.03 (-0.17, 0.11)	0.651	0.02 (-0.01, 0.05)	0.275	0.140
PFNA	-0.42 (-0.62, -0.23)	<0.001	-0.10 (-0.14, -0.06)	<0.001	<0.001
PFUA	0.04 (-0.15, 0.22)	0.700	-0.02 (-0.06, 0.01)	0.239	0.623

Alcohol	Yes		No		
	Estimate (95% CI)	P - value	Estimate (95% CI)	P - value	P - int
PFDA	-0.05 (-0.09, 0.00)	0.032	-0.04 (-0.12, 0.03)	0.279	0.768
PFOA	-0.27 (-0.31, -0.22)	<0.001	-0.34 (-0.41, -0.26)	<0.001	0.094
PFOS	-0.16 (-0.20, -0.12)	<0.001	-0.19 (-0.25, -0.12)	<0.001	0.504
PFHxS	-0.19 (-0.22, -0.15)	<0.001	-0.20 (-0.26, -0.14)	<0.001	0.789
N-MEFOSAA	0.00 (-0.03, 0.04)	0.781	0.02 (-0.04, 0.08)	0.540	0.886
PFNA	-0.11 (-0.16, -0.06)	<0.001	-0.19 (-0.27, -0.11)	<0.001	0.124
PFUA	-0.02 (-0.06, 0.02)	0.389	-0.01 (-0.09, 0.07)	0.764	0.596
BMI	<25		≥25		
	Estimate (95% CI)	P - value	Estimate (95% CI)	P - value	P - int
PFDA	-0.03 (-0.10, 0.04)	0.343	-0.05 (-0.10, -0.01)	0.025	0.975
PFOA	-0.25 (-0.32, -0.17)	<0.001	-0.30 (-0.35, -0.25)	<0.001	0.581
PFOS	-0.15 (-0.21, -0.08)	<0.001	-0.18 (-0.22, -0.14)	<0.001	0.967
PFHxS	-0.15 (-0.20, -0.09)	<0.001	-0.21 (-0.25, -0.17)	<0.001	0.304
N-MEFOSAA	-0.02 (-0.07, 0.04)	0.569	0.02 (-0.02, 0.05)	0.418	0.338
PFNA	-0.10 (-0.18, -0.03)	0.007	-0.15 (-0.20, -0.09)	<0.001	0.744
PFUA	-0.04 (-0.11, 0.03)	0.255	-0.01 (-0.06, 0.04)	0.631	0.311
Race	Non-Hispanic White		Other		
	Estimate (95% CI)	P - value	Estimate (95% CI)	P - value	P - int
PFDA	-0.05 (-0.10, 0.01)	0.082	-0.05 (-0.11, 0.01)	0.075	0.683
PFOA	-0.17 (-0.23, -0.12)	<0.001	-0.38 (-0.44, -0.32)	<0.001	<0.001
PFOS	-0.12 (-0.17, -0.07)	<0.001	-0.22 (-0.27, -0.17)	<0.001	0.004
PFHxS	-0.12 (-0.17, -0.08)	<0.001	-0.25 (-0.30, -0.21)	<0.001	<0.001
N-MEFOSAA	0.04 (0.00, 0.08)	0.055	-0.03 (-0.08, 0.01)	0.134	0.008
PFNA	-0.08 (-0.14, -0.03)	0.003	-0.19 (-0.25, -0.13)	<0.001	0.010
PFUA	-0.03 (-0.08, 0.02)	0.274	-0.01 (-0.06, 0.04)	0.704	0.841
Education	Less than high school		Other		
	Estimate (95% CI)	P - value	Estimate (95% CI)	P - value	P - int
PFDA	-0.05 (-0.11, 0.01)	0.083	-0.04 (-0.09, 0.01)	0.155	0.978
PFOA	-0.30 (-0.36, -0.23)	<0.001	-0.27 (-0.33, -0.22)	<0.001	0.594
PFOS	-0.17 (-0.22, -0.12)	<0.001	-0.17 (-0.21, -0.12)	<0.001	0.123
PFHxS	-0.20 (-0.25, -0.15)	<0.001	-0.17 (-0.21, -0.13)	<0.001	0.564
N-MEFOSAA	0.03 (-0.02, 0.07)	0.282	0.00 (-0.04, 0.04)	0.844	0.113
PFNA	-0.13 (-0.19, -0.07)	<0.001	-0.13 (-0.19, -0.07)	<0.001	0.496
PFUA	-0.01 (-0.07, 0.05)	0.788	-0.02 (-0.08, 0.03)	0.391	0.649

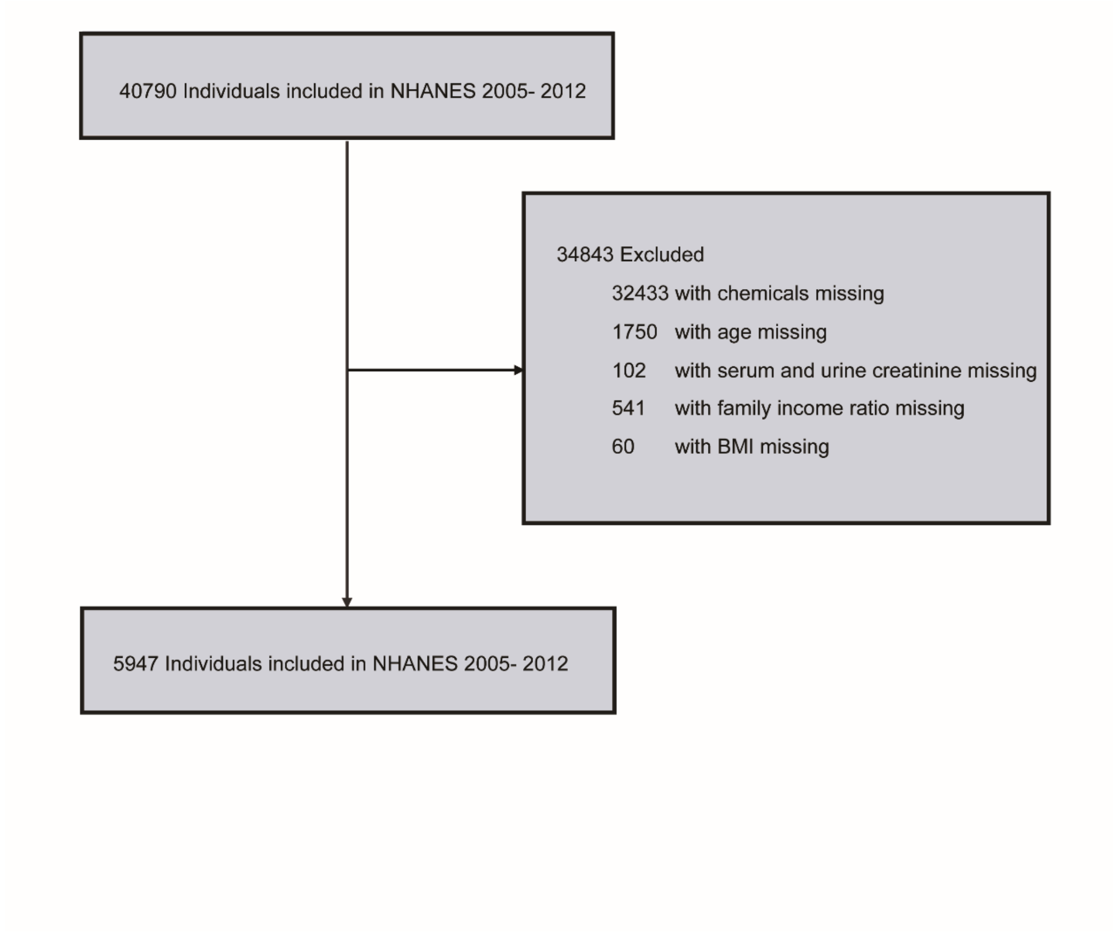

Fig. S1. Flowchart of inclusion and exclusion.

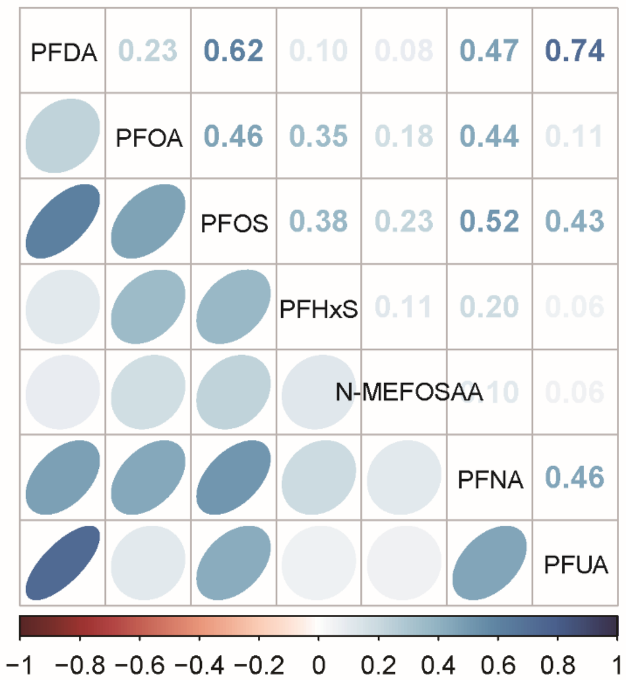

Fig. S2. Spearman correlation of PFAS.
